# Malaria-induced interferon-γ drives the expansion of Tbet^hi^ atypical memory B cells

**DOI:** 10.1371/journal.ppat.1006576

**Published:** 2017-09-27

**Authors:** Nyamekye Obeng-Adjei, Silvia Portugal, Prasida Holla, Shanping Li, Haewon Sohn, Abhijit Ambegaonkar, Jeff Skinner, Georgina Bowyer, Ogobara K. Doumbo, Boubacar Traore, Susan K. Pierce, Peter D. Crompton

**Affiliations:** 1 Malaria Infection Biology and Immunity Section, Laboratory of Immunogenetics, National Institute of Allergy and Infectious Diseases, National Institutes of Health, Rockville, Maryland, United States of America; 2 Center for Infectious Diseases, Parasitology, Heidelberg University Hospital, Heidelberg, Germany; 3 Lymphocyte Activation Section, Laboratory of Immunogenetics, National Institute of Allergy and Infectious Diseases, National Institutes of Health, Rockville, Maryland, United States of America; 4 The Jenner Institute Laboratories, University of Oxford, Oxford, United Kingdom; 5 Malaria Research and Training Centre, Department of Epidemiology of Parasitic Diseases, International Center of Excellence in Research, University of Sciences, Technique and Technology of Bamako, Bamako, Mali; Queensland Institute of Medical Research, AUSTRALIA

## Abstract

Many chronic infections, including malaria and HIV, are associated with a large expansion of CD21^−^CD27^−^ ‘atypical’ memory B cells (MBCs) that exhibit reduced B cell receptor (BCR) signaling and effector functions. Little is known about the conditions or transcriptional regulators driving atypical MBC differentiation. Here we show that atypical MBCs in malaria-exposed individuals highly express the transcription factor T-bet, and that T-bet expression correlates inversely with BCR signaling and skews toward IgG3 class switching. Moreover, a longitudinal analysis of a subset of children suggested a correlation between the incidence of febrile malaria and the expansion of T-bet^hi^ B cells. The Th1-cytokine containing supernatants of malaria-stimulated PBMCs plus BCR cross linking induced T-bet expression in naïve B cells that was abrogated by neutralizing IFN-γ or blocking the IFN-γ receptor on B cells. Accordingly, recombinant IFN-γ plus BCR cross-linking drove T-bet expression in peripheral and tonsillar B cells. Consistent with this, Th1-polarized Tfh (Tfh-1) cells more efficiently induced T-bet expression in naïve B cells. These data provide new insight into the mechanisms underlying atypical MBC differentiation.

## Author summary

Antibodies are proteins in blood that help kill microbes such as viruses, bacteria and parasites. Antibodies are produced by B cells with the help of T follicular helper (Tfh) cells. Some microbes for which we have no effective vaccines, such as HIV and malaria, establish chronic infections that are not cleared by the immune system. These chronic infections are associated with ‘atypical’ B cells that are less able to produce antibodies. We studied blood samples of malaria-exposed children to understand why normal B cells become atypical B cells. We found that atypical B cells express high levels of T-bet—a protein that is important for determining the fate of other types of immune cells. Children who frequently got malaria had more T-bet expressing B cells than children who rarely got malaria. We also found that malaria parasites cause immune cells to secrete inflammatory substances that cause normal B cells to express T-bet. Similarly, the inflammation-prone Tfh cells that malaria activates, which are relatively poor B cell helpers, also caused normal B cells to express T-bet. This study helps us understand why atypical B cells arise during chronic infections—information that could lead to strategies to improve antibody responses through vaccination.

## Introduction

Over the past decade it has become evident that many chronic infections and autoimmune diseases are associated with fundamental differences in the composition and functionality of B cell memory pools. [1] For example, the chronic infections HIV, malaria, and tuberculosis—that together cause more than 5 million deaths annually and continue to elude conventional vaccine development [2]—are all associated with an expansion of somatically hypermutated CD21^−^CD27^−^CXCR3^+^CD11c^+^ B cells that upregulate inhibitory receptors and exhibit decreased effector function [3–6], a subset of memory B cells (MBCs) that has been referred to as ‘atypical’ or ‘exhausted’. It has been postulated that atypical MBCs contribute to the humoral deficiencies associated with HIV and malaria, and may pose a challenge for the development of effective vaccines for these and other chronic infections. Despite the potential clinical relevance of atypical MBCs to the prevention and treatment of chronic infections and autoimmune diseases, little is known about the cellular and molecular mechanisms that drive their differentiation.

In the case of malaria, children generally mount short-lived antibody responses to *Plasmodium falciparum* infection, leaving them susceptible to repeated bouts of malaria [7]. Similarly, the only malaria vaccine candidate that has been tested in phase 3 clinical trials to date induces short-lived antibody responses [8, 9] and confers only partial, short-term protection against malaria in African children [10].

It is now well-established that long-lived humoral immunity depends on the activation of highly functional T follicular helper (Tfh) cells that support the differentiation of naive B cells into long-lived plasma cells (LLPCs) and MBCs in the germinal center (GC) reaction [11]. Although several Tfh subsets have been described in humans, data in healthy U.S. adults indicates that Th2-polarized, CXCR3-Tfh cells provide superior B cell help [12]. Consistent with the observation that malaria induces short-lived antibody responses, we recently observed that acute febrile malaria in children preferentially activates Th1-polarized PD-1^+^CXCR3^+^ Tfh (Tfh-1) cells that exhibit reduced B cell helper function [13], which is in line with several recent studies in mice showing that excessive IFN-γ suppresses germinal center B cell responses and anti-*Plasmodium* humoral immunity [14–17]. Taken together, these observations suggest that Th1 cytokines and Tfh-1 cells may play a role in the differentiation of atypical MBCs.

Here we conducted ex vivo analyses of immune cells of *P*. *falciparum*-exposed children in Africa, as well as in vitro modeling of malaria infection, to gain insights into the molecular and cellular conditions that are associated with the expansion of atypical MBCs.

## Results

### T-bet is highly expressed in atypical MBCs, correlates with reduced BCR signaling and skews toward IgG3 class switching

**Fig 1:**
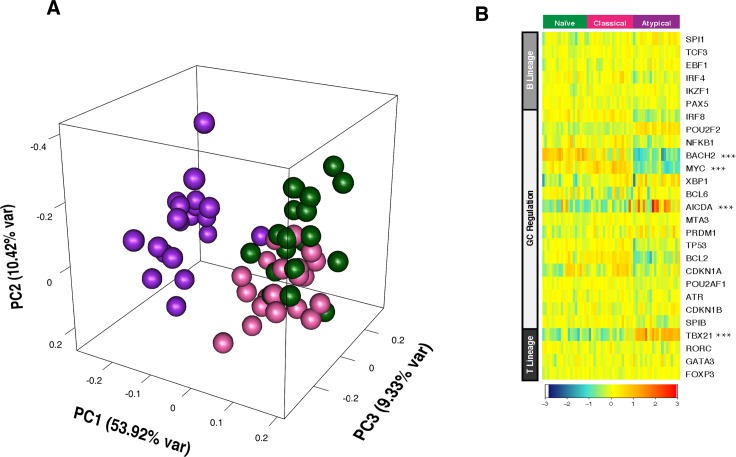
Malaria-associated atypical MBCs upregulate *TBX21*. (**A**) Principal components analysis of gene expression of selected regulators of B and T cell differentiation (selected genes shown in **B**) in naïve B cells (CD19^+^CD21^+^CD27^−^; green), classical MBCs (CD19^+^CD21^+^CD27^+^; pink) and atypical MBCs (CD19^+^CD21^−^CD27^−^; purple). (**B**) Ex vivo RMA-normalized log2 gene expression values of selected regulators of B and T cell differentiation (rows) for each subject (columns; n = 20) within each B cell subpopulation. Differentially expressed genes in atypical MBCs relative to classical MBCs are indicated with an asterisk.

**Fig 2:**
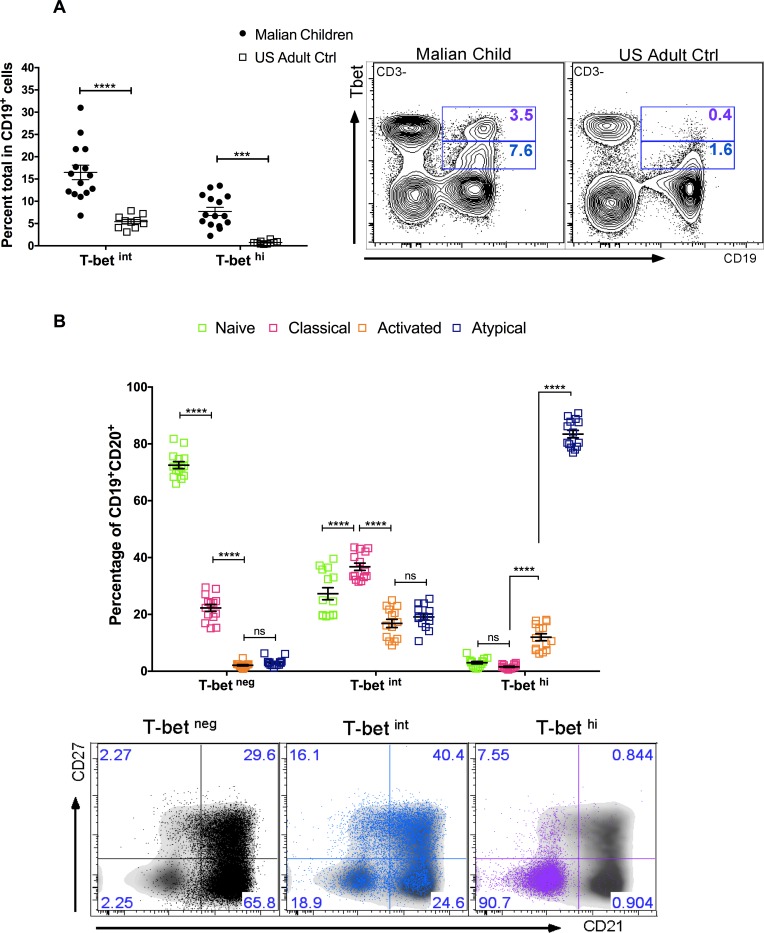
T-bet is highly expressed in malaria-associated atypical MBCs. **(A)** Ex vivo T-bet expression in total CD19^+^ B cells of representative subjects (right), and Malian children (n = 15) and U.S. adults (n = 10) (left). (**B**) Ex vivo distribution of T-bet^neg^, T-bet^int^ and T-bet^hi^ cells stratified by B cell subpopulations in Malian children (n = 15); representative FACS plot (bottom) shows T-bet^neg^ (black), T-bet^int^ (blue) and T-bet^hi^ (purple) cells. p values determined by paired Student’s *t* test with Bonferroni corrections for multiple comparisons where appropriate. ****P<0.0001, ***P<0.001, **P<0.01, *P<0.05, ns = not significant.

**Fig 3:**
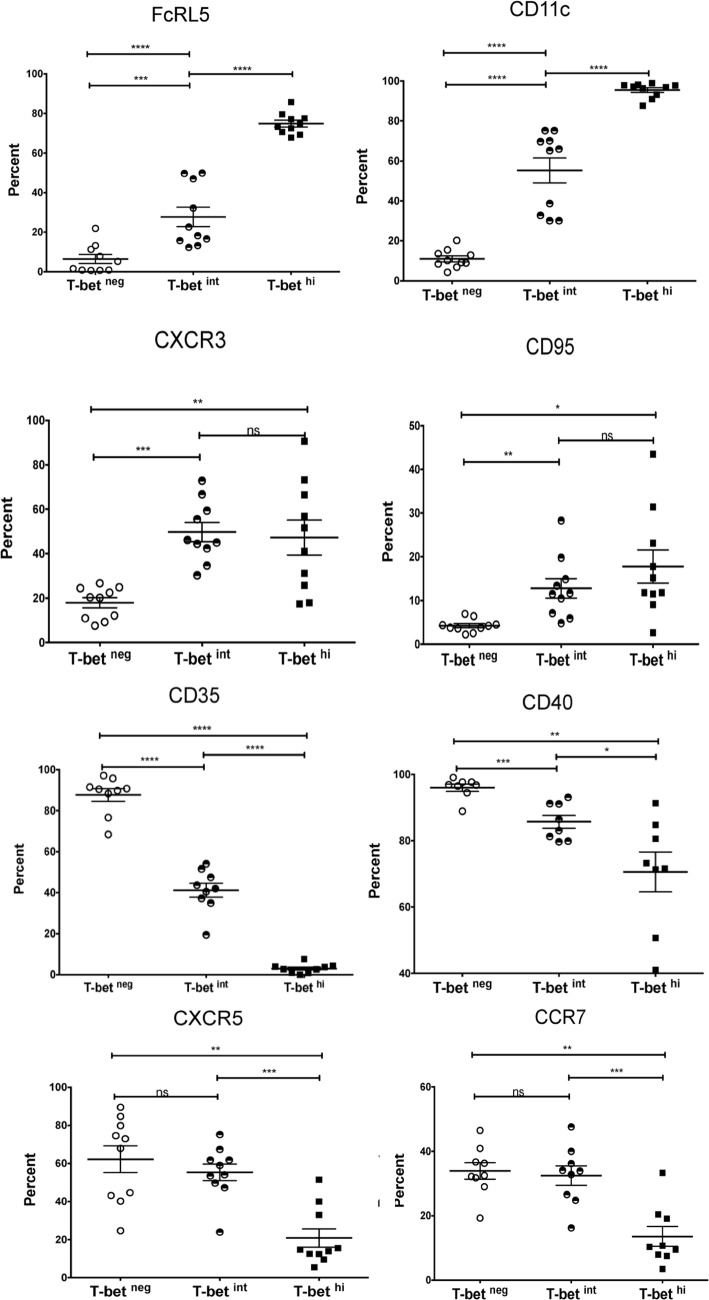
T-bet^hi^ B cells of malaria-exposed children express markers associated with atypical MBCs. Ex vivo expression of cell surface proteins on total CD19^+^ B cells stratified by level of T-bet expression in Malian children (n = 10). p values determined by paired Student’s *t* test with Bonferroni corrections for multiple comparisons where appropriate. ****P<0.0001, ***P<0.001, **P<0.01, *P<0.05, ns = not significant.

**Fig 4:**
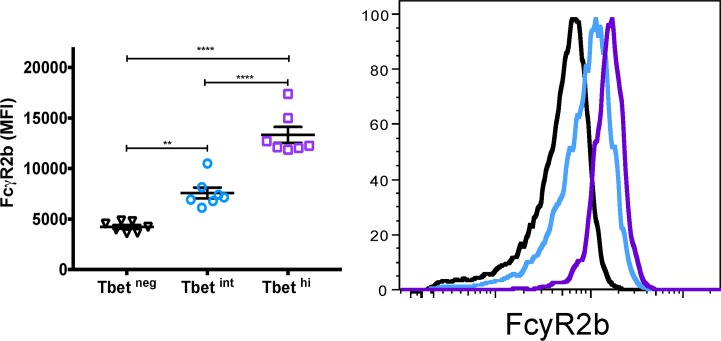
T-bet^hi^ B cells of malaria-exposed children upregulate the inhibitory receptor FcyR2B. Ex vivo expression of FcyR2B on total CD19^+^ B cells stratified by level of T-bet expression in Malian children (n = 7; representative subject, right). p values determined by paired Student’s *t* test with Bonferroni corrections for multiple comparisons where appropriate. ****P<0.0001, ***P<0.001, **P<0.01, *P<0.05, ns = not significant.

**Fig 5:**
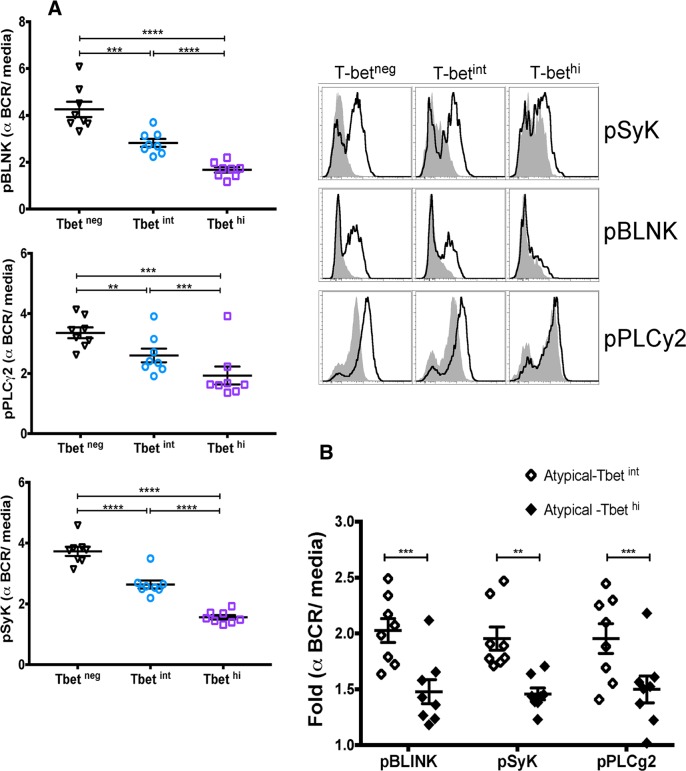
T-bet^hi^ atypical MBCs of malaria-exposed children exhibit reduced BCR signaling. (**A**) Ratio of phosphorylated Syk, BLNK, and PLCγ2 following BCR cross-linking over baseline fluorescence in negatively sorted B cells (n = 8 Malian children; representative subject, right). (**B**) MFI of pBLNK, pSyk and pPLCγ2 in T-bet^int^ and T-bet^hi^ atypical MBCs after BCR cross-linking (n = 8 Malian children). p values determined by paired Student’s *t* test with Bonferroni corrections for multiple comparisons where appropriate. ****P<0.0001, ***P<0.001, **P<0.01, *P<0.05, ns = not significant.

To gain insight into the regulators of atypical MBC differentiation we analyzed publicly available genome-wide expression data we generated from naïve B cells (CD19^+^CD21^+^CD27^−^), classical MBCs (CD19^+^CD21^+^CD27^+^) and atypical MBCs (CD19^+^CD21^−^CD27^−^) isolated from the peripheral blood of adults with lifelong malaria exposure (S1 Table) [5]. A principal components analysis (Fig 1A) of genes selected for involvement in lymphocyte differentiation and germinal center regulation (listed in Fig 1B) showed that atypical MBCs are transcriptionally distinct from classical MBCs. We found that relative to classical MBCs, atypical MBCs upregulate *TBX21* [fold change (FC) 2.7 (range 1.3–5.5), false discovery rate (FDR) adjusted p value = 1.008 E-10] and *AICDA* (FC 2.2, FDR p = 0.048), and downregulate *BACH2* (FC -2.1, FDR p = 2.733 E-07) and *MYC* (FC -2.5, FDR p = 1.549 E-15) (Fig 1B). *TBX21* encodes the Th1-lineage defining transcription factor T-bet, which we found is upregulated in B cells of malaria-exposed children (n = 15; S2 Table) relative to healthy U.S adults (n = 10) in a bi-modal distribution with approximately 18% of CD19^+^ B cells expressing intermediate levels of T-bet (T-bet^int^) and 8% expressing high levels of T-bet (T-bet^hi^) (Fig 2A). On average, atypical MBCs as a percentage of total B cells were 12.0% and 2.5% for Malian children and U.S. subjects, respectively. Among T-bet^hi^ B cells, 83.5% were atypical MBCs (95% CI: 80.6–86.3) and 12.0% were activated MBCs (95% CI: 9.3–14.6) (Fig 2B). Conversely, 79.8% of atypical MBCs (95% CI: 74.1–85.5) were T-bet^+^ and of these 63.3% were T-bet^hi^ (95% CI: 56.2–70.4). Moreover, in an independent experiment (n = 10 Malian children) T-bet^hi^ B cells of malaria-exposed children expressed markers that are known to be associated with atypical MBCs, with higher surface expression of FCRL5, CD11c, CXCR3 and CD95, and decreased expression of CD35, CD40, CXCR5 and CCR7 [5, 18] (Fig 3). Additionally, FCGR2B, a receptor known to reduce antibody production in B cells, was also upregulated in T-bet^hi^ B cells in an independent set of samples (n = 7 Malian children) (Fig 4). Consistent with this, T-bet^hi^ B cells exhibited lower phosphorylation of B cell receptor (BCR) signaling molecules following BCR cross-linking **(**Fig 5A)—a functional feature of atypical MBCs described previously.[5] Moreover, within CD21^-^CD27^-^ atypical MBCs, T-bet expression correlated inversely with phosphorylation of BCR signaling molecules (Fig 5B).

**Fig 6:**
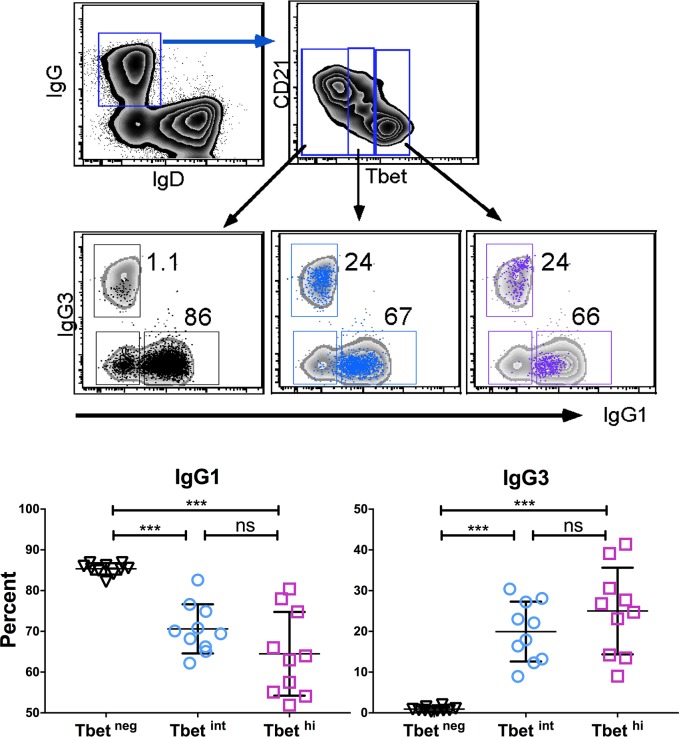
T-bet expression in B cells of malaria-exposed children associates with IgG3 class switching. Percentage of CD19+ B cells that express surface IgG1 or IgG3 stratified by level of T-bet expression (n = 9 Malian children; representative subject, top). p values determined by paired Student’s *t* test with Bonferroni corrections for multiple comparisons where appropriate. ****P<0.0001, ***P<0.001, **P<0.01, *P<0.05, ns = not significant.

**Fig 7:**
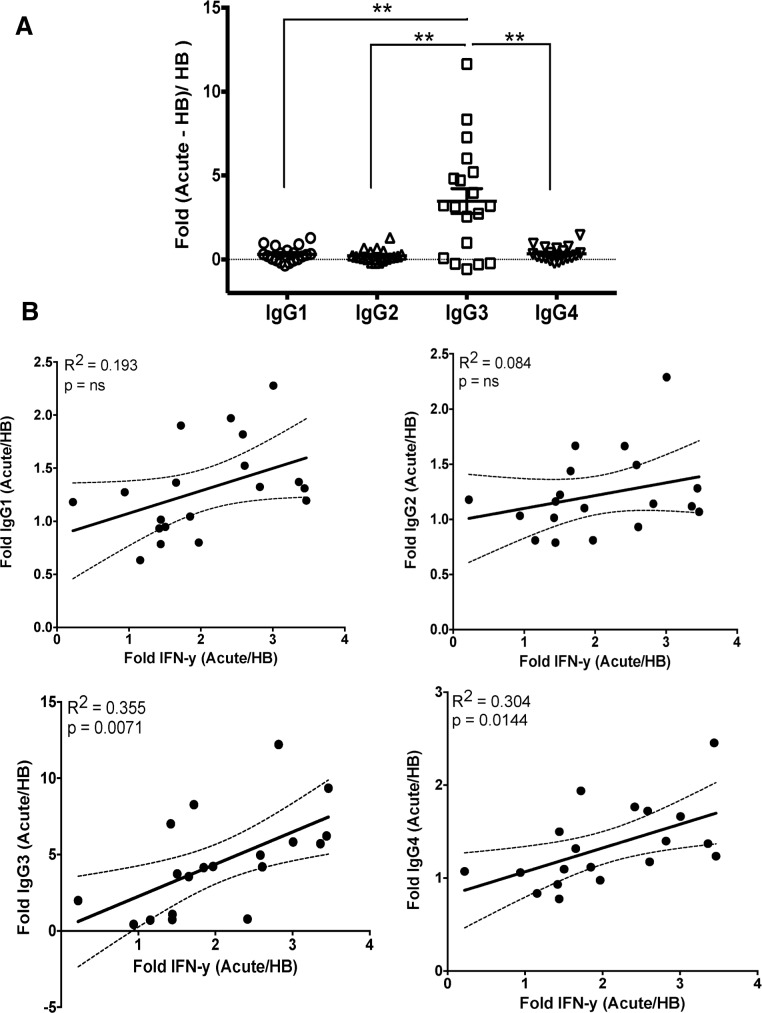
Change in IgG subclass levels during acute malaria and correlation with plasma IFN-γ levels. (**A**) Fold change in total plasma IgG subclasses during acute malaria relative to pre-infection baseline (n = 19 Malian children). (**B**) Fold change in total plasma IgG subclasses vs fold change in plasma IFN-γ during acute malaria relative to pre-infection baseline (n = 19 Malian children). p values determined by paired Student’s *t* test with Bonferroni corrections for multiple comparisons where appropriate. Paired Student’s *t* test and Pearson correlation were used for correlative analyses. ****P<0.0001, ***P<0.001, **P<0.01, *P<0.05, ns = not significant.

In mice, T-bet is known to be a selective inducer of IFN-γ-mediated IgG2a class switching in B cells.[19, 20] Therefore, we investigated the relationship between T-bet expression and isotype switching in B cells of Malian children. Overall, the percentage of IgG-expressing mature B cells was higher in Malian children compared to U.S. adults (S1 Fig). We found that B cells expressing intermediate or high levels of T-bet were more likely to surface express IgG3, compared to T-bet negative B cells which skewed toward IgG1 surface expression (Fig 6). Accordingly, T-bet expression (MFI) correlated with the percentage of IgG3^+^ B cells but not IgG1^+^ B cells (S2 Fig). In addition, total serum IgG3 had the greatest fold increase among IgG subclasses during acute malaria (Fig 7A) and (S3 Fig), which correlated with serum IFN-γ levels during the same malaria episode (Fig 7B).

Therefore, in malaria-exposed children, high T-bet expression appears to be a useful marker for atypical MBCs, as its correlates with phenotypic and BCR signaling properties of atypical MBCs. Moreover, T-bet expression in B cells of these children is associated with skewing toward IgG3 class switching.

### Febrile malaria induces Th1 cytokines and is associated with increased T-bet^hi^ B cells

**Fig 8:**
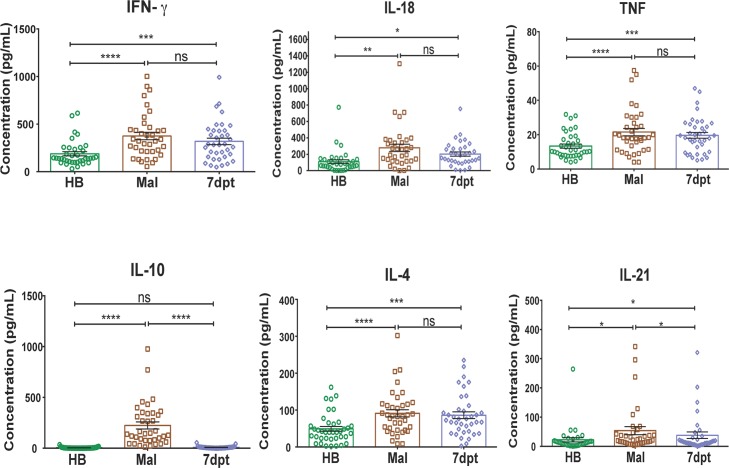
Plasma cytokine response in children during acute malaria. Plasma cytokine levels in Malian children (n = 37) at their healthy baseline (HB), during acute febrile malaria (Mal) and 7 days after anti-malarial treatment (7dpt). The Th2 cytokines IL-5 and IL-13 were not detectable at any time point. p values were determined by ANOVA with Sidak corrections where appropriate. ****P<0.0001, ***P<0.001, **P<0.01, *P<0.05, ns = not significant.

In a longitudinal analysis of serum collected from children at their healthy baseline before the malaria season, during acute febrile malaria, and 7 days post anti-malarial treatment, we confirmed in the present study population that acute febrile malaria tends to induce Th1-skewed cytokine production, with no IL-5 or IL-13 detected at any time point (Fig 8)—a response that has been associated with the activation of Tfh-1 cells that exhibit impaired B cell helper function in children [13] as well as germinal center dysfunction in *Plasmodium*-infected mice. [14–17]

**Fig 9:**
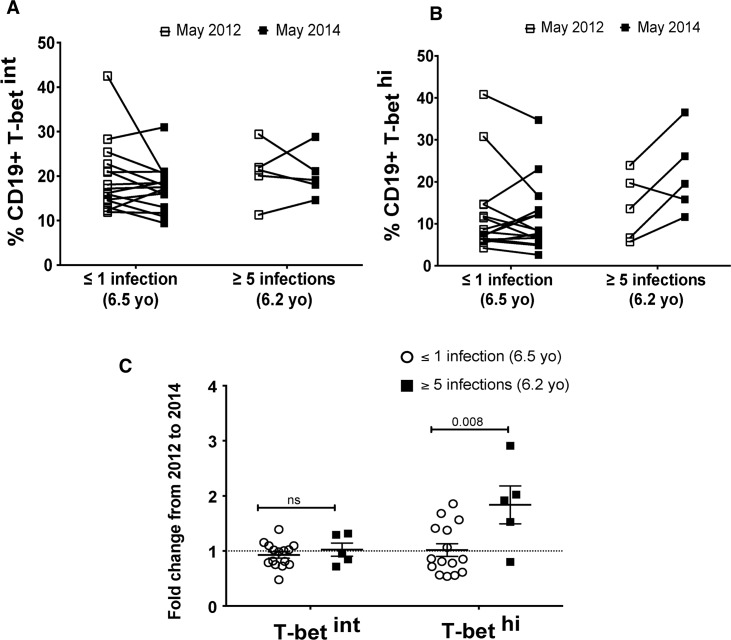
The frequency of febrile malaria episodes associates with increased T-bet^hi^ B cells. Percent of CD19^+^ B cells that were (**A**) T-bet^int^ or (**B**) T-bet^hi^ before and after a two-year period of intensive clinical surveillance in age-matched children who had either ≤1 (n = 15) or ≥5 (n = 5) febrile malaria episodes during the same period. (**C**) Fold change of T-bet^int^ and T-bet^hi^ CD19+ B cells from before to after the two-year surveillance period in children who had either ≤1 or ≥5 febrile malaria episodes. p values were determined by paired Student’s *t* test with Bonferroni adjustments where appropriate. ****P<0.0001, ***P<0.001, **P<0.01, *P<0.05, ns = not significant.

Because Th1 cytokines are known to drive T-bet expression in murine B cells [20, 21], we hypothesized that frequent febrile malaria episodes in children would be associated with an increase in T-bet^hi^ B cells. To test this hypothesis, we compared the change in T-bet expression in B cells in a subset of age-matched children who either had ≤1 or ≥5 febrile malaria episodes documented during two consecutive years of intensive clinical surveillance, and who had PBMCs available before and after the two-year surveillance period. Malaria episodes were defined as parasitemia of ≥2500 parasites/μL of blood, an axillary temperature of ≥37.5°C and no other cause of fever discernible by the study physician. We found that only children with ≥5 febrile malaria episodes had a significant increase in the percentage of T-bet^hi^ B cells from before to after the two-year period (Fig 9A–9C). Together these data suggest that malaria-induced Th1 cytokines drive T-bet expression in B cells, and thus play a role in the differentiation of T-bet^hi^ atypical MBCs.

### *P*. *falciparum*-induced IFN-γ plus BCR cross-linking drive T-bet expression in peripheral B cells

**Fig 10:**
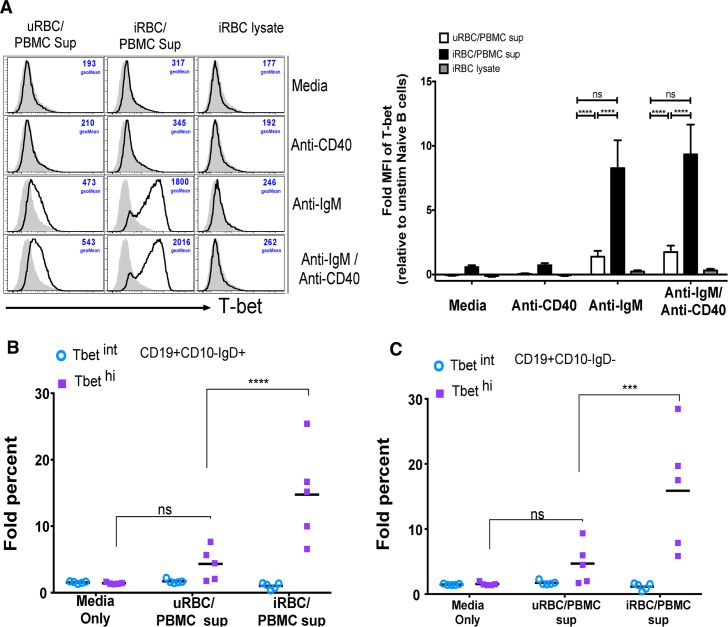
Supernatants of PBMCs stimulated with *P*. *falciparum*-infected RBCs plus BCR cross-linking drive T-bet expression in B cells. (**A-C**) PBMCs of healthy U.S. adults (n = 5) were stimulated in vitro with *P*. *falciparum*-infected red blood cell (iRBC) lysate or uninfected red blood cell (uRBC) lysate for 3 days. The resulting supernatants or the iRBC lysate alone were transferred to PBMCs from the same U.S. adults (n = 5) in the presence of media alone, anti-IgM, anti-CD40, or both, followed by staining for T-bet, CD10, CD19 and IgD. (**A**) Fold change in T-bet MFI in stimulated naïve B cells relative to unstimulated naïve B cells (left, representative histograms). Fold change in percentage of T-bet intermediate (T-bet^int^) and T-bet high (T-bet^hi^) (**B**) naïve B cells and (**C**) memory B cells after BCR cross-linking with anti-IgM/G/A in the presence of media alone, uRBC/PBMC supernatant or iRBC/PBMC supernatant, relative to unstimulated cells. Horizontal bars and whiskers represent means or median and SE. p values were determined by paired Student’s *t* test with Bonferroni adjustments where appropriate. ****P<0.0001, ***P<0.001, **P<0.01, *P<0.05, ns = not significant.

**Fig 11:**
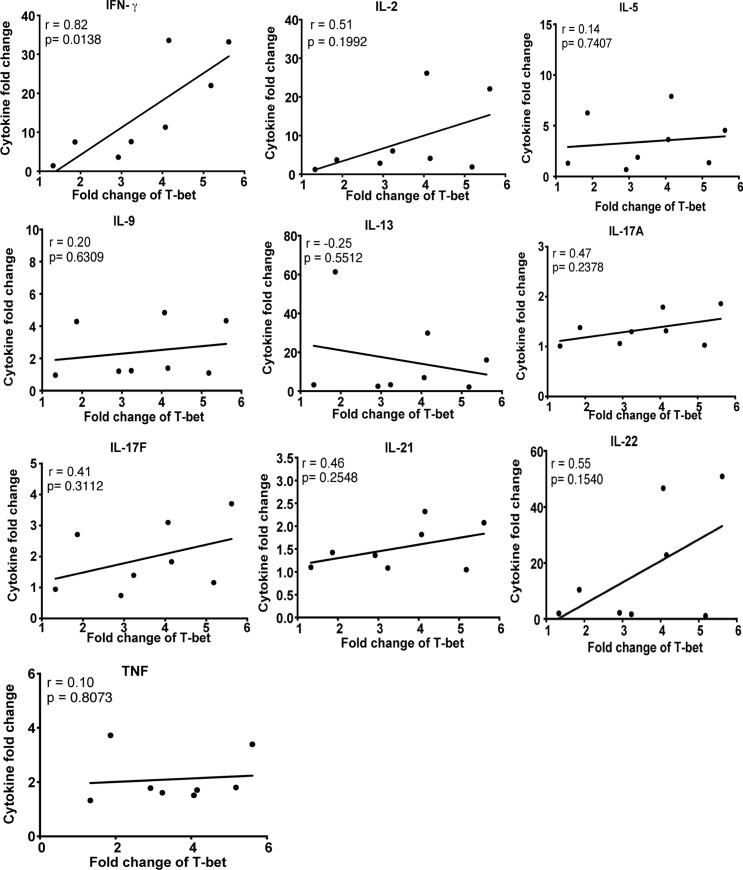
*P*. *falciparum*-induced IFN-γ correlates with T-bet expression in naïve B cells. PBMCs of healthy U.S. adults (n = 8) were stimulated in vitro with iRBC lysate or uRBC lysate for 3 days. Cytokine concentrations in the resulting supernatants were determined by a multiplex assay. Supernatants were transferred to PBMCs from the same U.S. adults (n = 8) in the presence of anti-IgM, followed by staining for T-bet, CD10, CD19 and IgD. Shown are correlations between fold changes in cytokine concentrations in supernatants of iRBC-stimulated vs. uRBC-stimulated PBMCs, and fold changes in T-bet expression in naïve B cells stimulated with supernatants of iRBC-stimulated vs. uRBC-stimulated PBMCs. Pearson correlation were used for correlative analyses. ****P<0.0001, ***P<0.001, **P<0.01, *P<0.05, ns = not significant.

**Fig 12:**
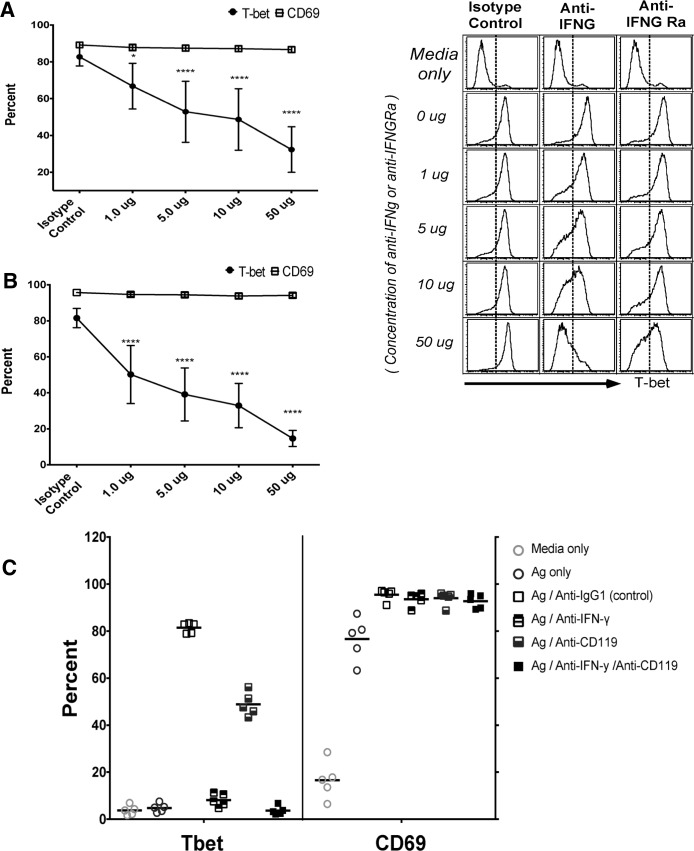
Blockade of *P*. *falciparum*-induced IFN-γ reduces T-bet expression in naïve B cells. T-bet and CD69 expression in naïve B cells (n = 5 U.S. adults) incubated with supernatants of iRBC-stimulated PBMCs plus anti-IgM in the presence of increasing concentrations of IFN-γ neutralizing antibodies (**A**) or IFN-γ receptor blocking antibodies (**B**) (representative histograms, right). (**C**) T-bet and CD69 expression in naïve B cells (n = 5 U.S. adults) incubated with supernatants of iRBC-stimulated PBMCs plus anti-IgM in the presence of IFN-γ neutralizing antibodies and IFN-γ receptor blocking antibodies separately or combined. Horizontal bars and whiskers represent means or median and SE. p values were determined by paired Student’s *t* test with Bonferroni adjustments where appropriate. ****P<0.0001, ***P<0.001, **P<0.01, *P<0.05, ns = not significant.

**Fig 13:**
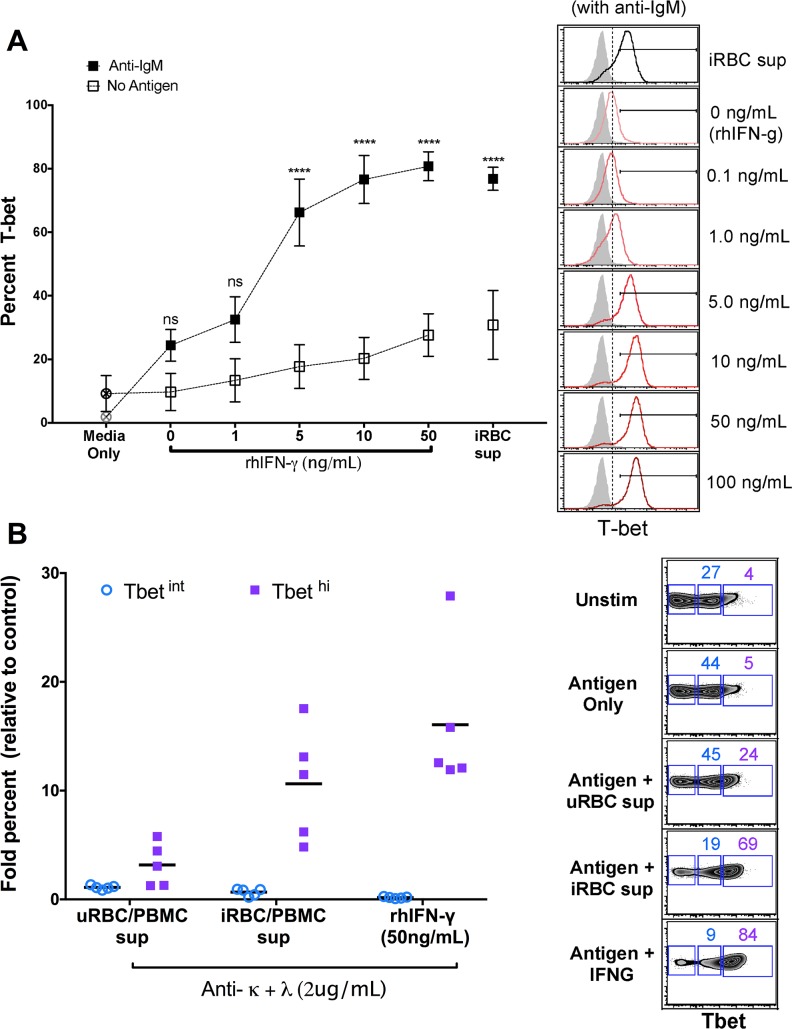
T-bet expression in naïve B cells in the presence of supernatant of iRBC-stimulated PBMCs or increasing concentrations of IFN-γ with or without BCR crosslinking. (**A**) T-bet expression in naïve B cells (n = 5 U.S. adults) with or without BCR crosslinking in the presence of supernatant of iRBC-stimulated PBMCs, or in the presence of increasing concentrations of recombinant human IFN-γ (right, representative histograms). (**B**) Intermediate and high T-bet expression in naïve B cells (n = 5 U.S. adults) after BCR cross-linking in the presence of iRBC-stimulated PBMC supernatant or rhIFN-γ (right, representative histograms). Horizontal bars and whiskers represent means or median and SE. p values were determined by paired Student’s *t* test with Bonferroni adjustments where appropriate. ****P<0.0001, ***P<0.001, **P<0.01, *P<0.05, ns = not significant.

To test the hypothesis that *P*. *falciparum*-induced cytokines drive T-bet expression in B cells we simulated malaria infection in vitro by co-culturing PBMCs of healthy U.S. donors with *P*. *falciparum*-infected red blood cells (iRBCs). The resulting supernatant induced T-bet expression in purified naïve B cells but only in the presence of BCR cross-linking by anti-IgM antibodies (Fig 10A), whereas exposure of purified naïve B cells to iRBC lysate alone in the presence of BCR cross-linking did not induce T-bet expression (Fig 10A). A similar pattern was observed for the activation markers CD69 and CD86 on naïve B cells (S4A and S4B Fig). Similar fold increases in T-bet^hi^ B cells were observed in both naïve and memory B cells (Fig 10B and 10C). Using cytokine multiplex assays, we measured 20 cytokines in the supernatants of U.S. PBMCs stimulated with uninfected RBCs (uRBCs) or iRBCs. Of the cytokines detected, ten (IFN-γ, IL-2, IL-5, IL-9, IL-13, IL-17A, IL-17F, IL-21, IL-22 and TNF) were significantly higher in the supernatants of iRBC-stimulated PBMCs (S5 Fig and S6 Fig). However, only the concentration of IFN-γ in the supernatant of PBMCs stimulated with iRBC lysate correlated with T-bet expression in naïve B cells treated with these supernatants plus anti-IgM (Fig 11). Accordingly, neutralization of IFN-γ in the supernatant or blocking IFN-γ Ra on B cells reduced T-bet expression in naïve B cells, but did not diminish expression of the activation marker CD69 (Fig 12A and 12B). Of note, combining antibodies that neutralize IFN-γ or block IFN-γ Ra in the same experiment further reduced the percentage of T-bet-expressing B cells to ~4% (Fig 12C). Consistent with this, BCR cross-linking plus recombinant human IFN-γ (rhIFN-γ) induced T-bet expression in a dose dependent manner and at a concentration of IFN-γ that was similar to that found in supernatants of iRBC-stimulated PBMCs (Fig 13A). While BCR stimulation alone drove intermediate T-bet expression in B cells, high T-bet expression was only observed with the addition of supernatants of iRBC-stimulated PBMCs or rhIFN-γ (Fig 13B).

Taken together, these data show that *P*. *falciparum*-induced IFN-γ drives T-bet expression in B cells through the IFN-γ receptor, consistent with the observation that repeated febrile malaria episodes are associated with increased T-bet^hi^ B cells.

### IFN-γ plus BCR cross-linking drive T-bet expression in tonsillar B cells

**Fig 14:**
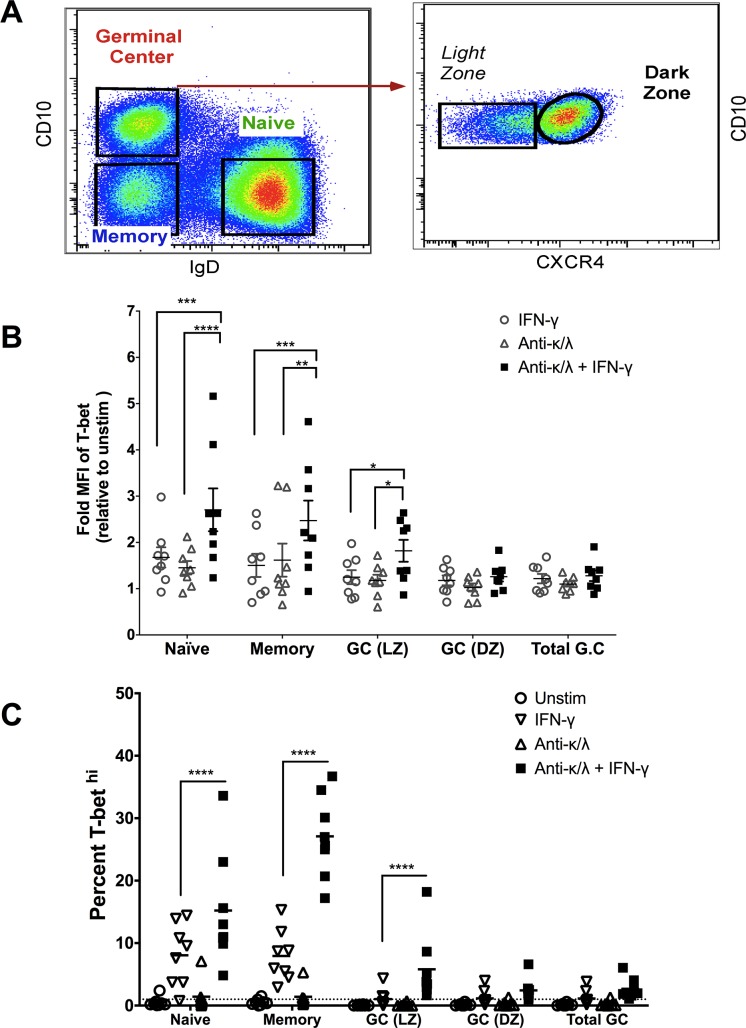
IFN-γ plus BCR crosslinking induce T-bet expression in tonsillar naïve B cells, MBCs and light zone germinal center B cells. B cell subsets were negatively selected from tonsillar tissue of U.S. children (n = 8) and gated on naïve B cells (CD10-, IgD+), MBCs (CD10-IgD-) and germinal center (GC) (CD10+IgD-) B cells. GC B cells were further stratified on light (CXCR4-) and dark (CXCR4+) zone GC B cells. Gating strategy shown in (**A**). (**B**) MFI of total T-bet and **(C)** percent T-bet^hi^ determined by FACS in B cell subsets following stimulation with rhIFN-γ, BCR cross-linking or both. p value were determined by 2 way ANOVA with Sidak corrections. ****P<0.0001, ***P<0.001, **P<0.01, *P<0.05, ns = not significant.

Next, we examined the effect of IFN-γ plus BCR cross-linking on T-bet expression in tonsillar B cell subsets of U.S. children. Consistent with our findings in peripheral B cells, we found that rhIFN-γ plus BCR cross-linking induced T-bet expression in naïve B cells (CD10^-^, IgD^+^), MBCs (CD10^-^, IgD^-^) and light zone GC B cells (CD10^+^IgD^-^CXCR4^-^) (Fig 14A–14C), whereas rhIFN-γ or BCR cross-linking alone did not significantly induce T-bet expression.

### Tfh-1 and Th1 cells more efficiently drive T-bet expression in naïve B cells

**Fig 15:**
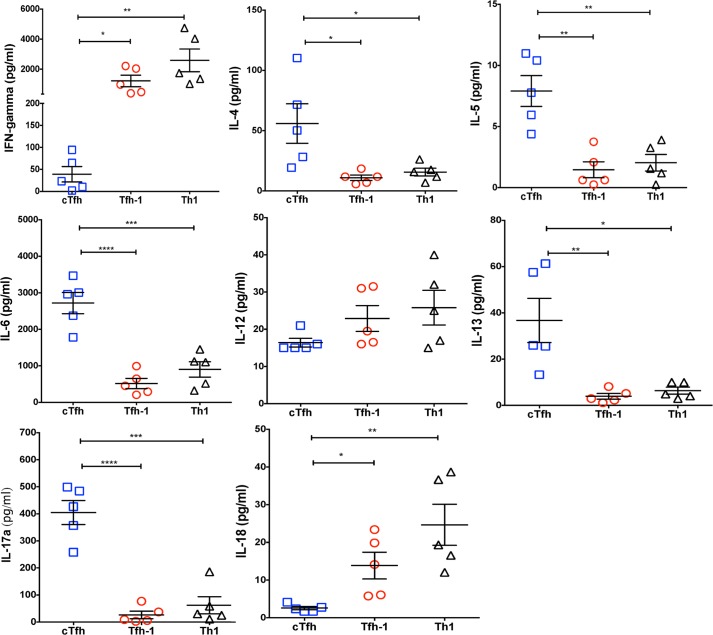
Secreted cytokine profiles of T cell subset/naïve B cell co-cultures. PBMCs of U.S. adults (n = 5) were FACs-sorted into naïve B cells (CD19+CD21+IgD+), cTfh cells (CD4+PD-1+CXCR5+CXCR3-), Tfh-1 cells (CD4+PD-1+CXCR5+CXCR3+) and Th-1 cells (CD4+PD-1+CXCR5-CXCR3+). Autologous naïve B cells were cultured for 2 days with each T cell subset in the presence of SEB, or with SEB alone. Shown are cytokine concentrations in supernatants at 2 days. p values were determined by paired Student’s *t* test with Bonferroni adjustments where appropriate. ****P<0.0001, ***P<0.001, **P<0.01, *P<0.05, ns = not significant.

**Fig 16:**
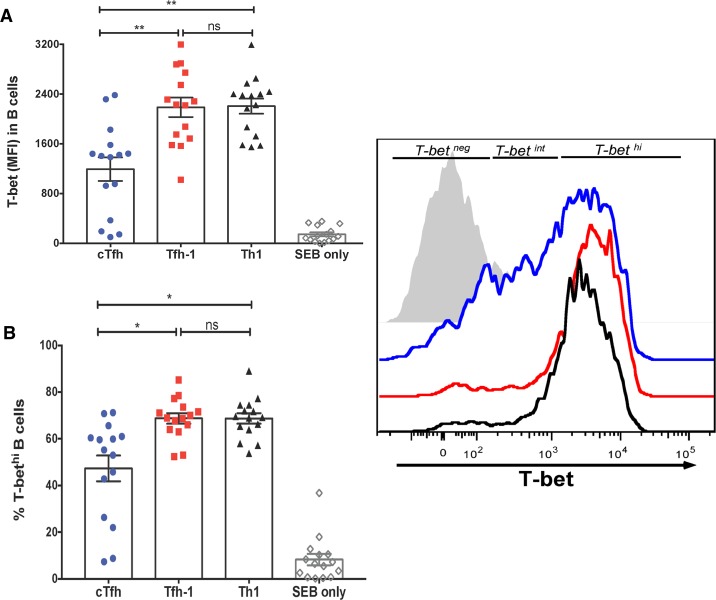
Tfh-1 and Th1 cells are superior inducers of T-bet expression in naïve B cells. PBMCs of U.S. adults (n = 15) were FACs-sorted into naïve B cells (CD19+CD21+IgD+), cTfh cells (CD4+PD-1+CXCR5+CXCR3-), Tfh-1 cells (CD4+PD-1+CXCR5+CXCR3+) and Th-1 cells (CD4+PD-1+CXCR5-CXCR3+). Autologous naïve B cells were cultured for 2 days with each T cell subset in the presence of SEB, or with SEB alone. (**A**) T-bet MFI in B cells at 2 days of co-culture with T cell subsets or SEB alone. Representative histogram on right. (**B**) Percentage of T-bet^hi^ B cells at 2 days of co-culture with T cell subsets or SEB alone. p values were determined by paired Student’s *t* test with Bonferroni adjustments. ****P<0.0001, ***P<0.001, **P<0.01, *P<0.05, ns = not significant.

Because febrile malaria in children has been shown to preferentially activate a subset of circulating Th1-polarized Tfh (Tfh-1) cells [13], we tested the hypothesis that Tfh-1 cells drive T-bet expression in B cells. We FACS-sorted PBMCs from healthy U.S. adults into naïve B cells (CD19+CD21+IgD+), Th-2 polarized circulating Tfh (cTfh) cells (CD4^+^PD^-^1^+^CXCR5^+^CXCR3^-^), Tfh-1 cells (CD4^+^PD-1^+^CXCR5^+^CXCR3^+^) and Th-1 cells (CD4^+^PD-1^+^CXCR5^-^CXCR3^+^) (gating strategy shown in S7 Fig). Autologous naïve B cells were cultured with each T cell subset in the presence of the super antigen staphylococcal enterotoxin B (SEB), or with SEB alone. We observed no difference in the percentage of viable B cells among the three co-culture conditions at day 2 (S8A Fig). In each condition after 2 days in culture, naïve B cells significantly upregulated the activation marker CD69 (S8B Fig) and Blimp-1 (S8C Fig) as well as AID to varying degrees (S8D Fig). As expected, the secreted cytokine profile in supernatants after 2 days was Th1-skewed in the presence of Th1 and Tfh-1 cells, and Th2/Th17-skewed in the presence of cTfh cells (Fig 15). Accordingly, a higher percentage of T-bet^hi^ B cells were detected when cultured with Tfh-1 and Th1 cells, compared to B cells cultured with cTfh cells or SEB alone (Fig 16A and 16B). The addition of rhIFN-γ to the naïve B cell/T cell subset culture increased the percentage of T-bet expressing B cells (S9 Fig). As expected, Tfh-1 cells, which more efficiently drove T-bet expression in B cells, were less efficient in driving autologous naïve B cells to class switch (S10A Fig) or produce antibodies (S10B Fig).

## Discussion

Several chronic infections are associated with an expansion of ‘atypical’ or ‘exhausted’ MBCs that upregulate inhibitory receptors and exhibit decreased effector function [3–5]. Here we conducted ex vivo analyses of immune cells of malaria-exposed children in Africa as well as in vitro studies to gain insight into the cellular and molecular conditions associated with the differentiation of atypical MBCs.

In malaria-exposed children we found that T-bet^hi^ B cells express markers that are known to be associated with atypical MBCs (FCRL5, FCGR2B, CD11c, CXCR3 and CD95) [5, 18]. Consistent with this, T-bet^hi^ B cells exhibited lower phosphorylation of BCR signaling molecules following BCR cross-linking—a functional feature of atypical MBCs described previously.[5] Moreover, within CD21^-^CD27^-^ atypical MBCs, T-bet expression correlated inversely with phosphorylation of BCR signaling molecules. Together these data suggest that atypical MBC differentiation occurs along a spectrum in which altered expression of key transcription factors drives the hierarchal upregulation and co-expression of inhibitory receptors (e.g. FCRL5, FCGR2B), which in turn leads to progressive loss of BCR signaling and effector functions—potentially analogous to what has been described for T cell exhaustion.[22]

We found that B cells that express T-bet at intermediate or high levels were more likely to surface-express IgG3, compared to T-bet negative B cells which skewed toward IgG1 expression. Moreover, total serum IgG3 had the greatest fold increase among IgG subclasses during acute malaria, which correlated with serum IFN-γ levels. Although differences in IgG subclasses between mice and humans make direct comparisons difficult, in mice, T-bet is a selective inducer of IFN-γ-mediated class switching to IgG2a [19, 20, 23], which is functionally similar to human IgG1 and IgG3 in terms of FcR binding and complement fixation capacity. Interestingly, a recent study showed that HIV infection drives the expansion and maintenance of T-bet^+^ B cells (discussed further below) that correlate with an overrepresentation of surface-expressed and soluble IgG1 and IgG3. [24] Therefore, there may be a consistent theme in mice and humans: that IFN-γ drives T-bet expression in B cells, which promotes class switching to IgG subclasses that are potent triggers of effector mechanisms. Further human studies are needed to determine the generalizability of these findings in other settings, and the mechanisms by which this occurs. However, based on these data we speculate that in the context of pediatric malaria, intermediate T-bet expression contributes to IgG3 class switching, while T-bet ‘overexpression’ may play a role in atypical MBC differentiation. It will also be of interest to investigate the potential role of intermediate T-bet expression in B cell activation given that the expression of CXCR3 and CD95 appears similar in T-bet intermediate and T-bet high B cells (Fig 3).

A preliminary longitudinal analysis of a subset of children suggested a correlation between the incidence of febrile malaria and the expansion of T-bet^hi^ B cells. Consistent with the Th1-skewed cytokine response that febrile malaria induces, we found that Th1-cytokine-containing supernatants of iRBC-stimulated PBMCs plus BCR crosslinking induced T-bet expression in naïve B cells, a response that was abrogated by neutralizing IFN-γ or blocking the IFN-γ receptor. Accordingly, recombinant human IFN-γ plus BCR cross-linking drove T-bet expression in peripheral B cells. Importantly, the same conditions drove T-bet expression in B cells derived from secondary lymphoid tissue (tonsils) where germinal center reactions occur.

Because Tfh cells are known to play a critical role in the activation and differentiation of naïve B cells in secondary lymphoid tissue [25], we also examined T-bet expression in naïve B cells following co-culture with various T cell subsets. Our previous studies confirmed that functionally distinct memory Tfh cell subsets can be detected in the circulation of malaria-exposed children [13, 26]. Additionally, we found previously that Th-1 polarized Tfh cells (Tfh-1) (T-bet^+^, IFN-γ-producing) that exhibit impaired B cell helper function are preferentially activated during acute febrile malaria in children [13]. In the present study, we observed higher T-bet expression in naïve B cells co-cultured with autologous Tfh-1 and Th1 cells, which produced high levels of IFN-γ and IL-18, consistent with the observation that IFN-γ drives T-bet expression in naïve B cells. Whereas Tfh-1 and Th1 cells primarily induce T-bet^hi^ B cells, intriguingly, Th-2 polarized cTfh cells, which produce much less IFN-γ, appear capable of inducing both intermediate and high T-bet expression in naïve B cells, albeit at lower levels, suggesting additional mechanism by which cTfh cells may induce T-bet in B cells. Because malaria can induce IFN-γ production in multiple cell types, it remains possible that IFN-γ from sources other than Tfh cells could drive T-bet expression in B cells in vivo. However, because of the proximity of Tfh cells and B cells in secondary lymphoid tissue, it seems plausible that Tfh-derived IFN-γ may play a greater role in driving T-bet expression in B cells. Together these data support the hypothesis that malaria-induced activation of Tfh-1 cells contributes to the expansion of T-bet^hi^ atypical MBCs in malaria-exposed children.

In general, little is known about T-bet^+^ B cells in humans [27]. In healthy adults T-bet has been detected in memory B cells and plasmablasts, but at lower levels than other T-bet^+^ lymphocytes.[28] T-bet expression in circulating CD21^-^CD27^-^ ‘tissue-like’ MBCs has been described in healthy adults, in whom CD21^-^CD27^-^ B cells are a relatively rare population [24, 29], but whether CD21^-^CD27^-^ B cells in healthy adults represent the same population of CD21^-^CD27^-^ atypical MBCs that are expanded in disease settings remains unclear. Interestingly, a recent study in humans showed that yellow fever and vaccinia vaccination stimulates an acute T-bet^+^ B cell response and that the T-bet^hi^CD85j^hi^ population may function as an early responder during acute viral infections. [24] Of note, the same study reported that HIV infection maintains an expanded T-bet^+^ B cell population that was primarily comprised of T-bet^hi^CD85j^hi^ B cells. [24]

Several recent studies have described T-bet expression in B cells of individuals with autoimmune diseases. For example, transcriptome analysis of CD21^-/lo^ versus CD21^+^ mature naïve B cells from subjects with rheumatoid arthritis or common variable immunodeficiency found that *TBX21* expression was upregulated in CD21^-/lo^ B cells [30]. Similarly, transcriptome analysis of CD19^+^ B cells isolated from individuals with systemic lupus erythematosus revealed increased *TBX21* expression compared to CD19^+^ B cells of healthy controls.[31] Importantly, HIV and malaria-associated atypical MBCs exhibit markedly reduced cytokine and antibody production capacity [4, 5, 32], whereas T-bet^+^ CD19^+^ B cells in individuals with autoimmune diseases can produce proinflammatory cytokines and autoreactive antibodies [33–35]. Therefore, T-bet^+^ B cells that arise in humans in the context of chronic infections versus autoimmunity may differ phenotypically and functionally, although further studies are needed to determine if this is a consistent pattern.

That IFN-γ drives T-bet expression in activated human B cells is consistent with prior studies in mouse models [20, 21, 36]. T-bet expressing B cells termed age-associated B cells (ABCs) appear in mice with age, autoimmunity and viral infections [37][38, 39]. ABCs are generated through the interplay of IL-4, IL-21, and IFN-γ in concert with TLR engagement [40], and have been shown to play a role in the pathogenesis of lupus-like autoimmunity [39] and anti-viral immunity [41, 42]. Although murine ABCs are similar to human atypical MBCs in that they upregulate T-bet and CD11c, and downregulate CD21, unlike atypical MBCs [5], murine ABCs proliferate in response to TLR agonists, produce IL-10 and IFN-γ and differentiate into ASCs—distinct functional profiles that call into question the relatedness of murine ABCs and human atypical MBCs that are associated with chronic infections. Instead, the available evidence suggests that murine ABCs more closely resemble the phenotype and function of T-bet^+^ B cells in humans with autoimmune diseases [31, 33, 35, 43].

It will be of interest in future studies to employ methods such as siRNA gene silencing and ChIP-seq to determine whether T-bet plays a causal role in atypical MBC differentiation, and if so, how it directly affects B cell programming, and what other transcription factors may be involved [44]. For example, we found that expression of the gene encoding the transcription factor Bach2—which predisposes GC B cells to enter the memory pool [45, 46]—was downregulated in atypical MBCs (Fig 1B).

A high priority should also be placed on ascertaining the ‘plasticity’ [47] of atypical MBCs, and whether and how their apparent loss of function can be reversed. In this regard, Kardava et al showed that HIV-associated human B cell exhaustion could be attenuated by siRNA downregulation of inhibitory receptors, particularly Fc receptor-like-4 (FCRL4) and sialic acid-binding Ig-like lectin 6 (Siglec-6) [32]. However, emerging data suggests that the array of inhibitory receptors expressed by atypical MBCs varies by disease; for example, malaria-associated atypical MBCs upregulate the expression of FCRL3 and FCRL5 rather than FCRL4 [5].

In summary, we show that atypical MBCs in malaria-exposed individuals highly express T-bet, and that exposure to malaria-induced Th1 cytokines and Tfh-1 cells correlates with the expansion of T-bet^hi^ B cells. These data provide insight into the mechanisms underlying atypical MBC differentiation and open the possibility of preventing or reversing the expansion of atypical MBCs in various disease states.

## Materials and methods

### Ethics statement

The Ethics Committee of the Faculty of Medicine, Pharmacy and Dentistry at the University of Sciences, Technique and Technology of Bamako, and the Institutional Review Board of the National Institute of Allergy and Infectious Diseases, National Institutes of Health approved this study. Written informed consent was obtained from participants or parents or guardians of participating children prior to inclusion in the Mali study. Human pediatric tonsil tissue was obtained from the pathology department at the Children's National Medical Center in Washington, DC, and written informed consent was obtained from the parents or legal guardians of all donors. All collected tonsil samples were anonymized.

### Mali study subjects and field site

The field study was conducted in the rural village of Kalifabougou, Mali where intense *P*. *falciparum* transmission occurs from June through December each year. The cohort study has been described in detail elsewhere [48]. Briefly, 695 healthy children and adults aged 3 months to 25 years were enrolled in an ongoing cohort study in May 2011. Exclusion criteria at enrollment included a hemoglobin level <7 g/dL, axillary temperature ≥37.5°C, acute systemic illness, underlying chronic disease, or use of antimalarial or immunosuppressive medications in the past 30 days. The present study focused on 74 children aged 3–12 years who had venous blood samples collected at their healthy uninfected baseline before the malaria season, as well as during and 7 days after treatment of their first acute malaria episode of the ensuing 6-month malaria season. Clinical malaria episodes were detected through active and passive surveillance and were defined as ≥ 2,500 asexual parasites/μL, an axillary temperature of ≥37.5°C and no other cause of fever discernible by physical exam. All individuals with signs and symptoms of malaria and any level of parasitemia detected by microscopy were treated according to the Malian National Malaria Control Program guidelines.

### U. S. donors

Peripheral blood samples from healthy U.S. adult donors enrolled in NIH protocol # 99-CC-0168) were also analyzed. Fresh human tonsils were obtained from the pathology department of the Children's National Medical Center in Washington, DC. All tonsils were from children. Demographic and travel history data were not available from these anonymous donors, but prior *P*. *falciparum* exposure is unlikely.

### Detection and quantification of *P*. *falciparum* infection

Thick blood smears were stained with Giemsa and counted against 300 leukocytes. Parasite densities were recorded as the number of asexual parasites per microliter of blood based on a mean leukocyte count of 7500 cells/μL.

### Sample processing

Mali blood samples (8 ml) were drawn by venipuncture into sodium citrate-containing cell preparation tubes (BD, Vacutainer CPT Tubes) and transported 45 km to the laboratory where PBMCs and plasma were isolated and frozen within three hours according to the manufacturer's instructions. Plasma was frozen at −80°C. PBMCs were frozen in fetal bovine serum (FBS) (Gibco, Grand Island, NY) containing 7.5% dimethyl sulfoxide (DMSO; Sigma-Aldrich). U.S. blood samples were drawn into heparinized tubes (BD) and PBMCs were isolated from whole blood by Ficoll-Hypaque density gradient centrifugation (GE Healthcare, Uppsala, Sweden) according to the manufacturer's instructions, and frozen under the same conditions as the Malian samples. For all assays, PBMCs were rapidly thawed in a 37°C water bath, washed in PBS with 10% heat-inactivated FBS and then in complete RPMI (RPMI 1640 with L-glutamine supplemented with 10% heat-inactivated FBS, and penicillin/streptomycin 10,000 μg/ml, [all from GIBCO, Invitrogen]). Tonsils were homogenized using wire mesh and passed through a cell strainer to make a single cell suspension. B cells were then negatively selected using a human B cell enrichment kit (STEMCELL Technologies).

### Microarray data analysis

Previously published gene expression microarray data [5] was re-analyzed to examine the expression of genes involved in lymphocyte differentiation and germinal center regulation in naïve, classical MBCs and atypical MBCs using R. Expression data were imported from.CEL files using the read.celfiles() command from the oligo package library [49] and normalized using robust multi-array average (RMA) [50]. Samples were checked for quality using density and principal components analysis (PCA) plots, which confirmed the presence of a previously identified outlier sample was removed from the analysis. The data were then median normalized and filtered to remove any low information probes that were not on the list of selected genes or any genes highlighted in the previous publication [5]. Probes were considered “low information” if they had mean log2 expression less than 5 or log2 expression standard deviation below 1. After normalization and filtering, a 3-dimensional PCA plot was created using the default settings of the prcomp() analysis in R. Differential expression of naïve B cells (N), classical MBCs (C) and atypical MBCs (A) was tested using limma [51], then heatmaps were generated using heatmap.2() from the gplots package library[52].

### Flow cytometry and cell sorting

For surface staining and sorting, PBMCs were washed in PBS with 4% heat-inactivated FCS and cells were incubated with live/dead fixable stain (Invitrogen) and the indicated fluorescently labeled antibodies. Cells stained for sorting were kept on ice until sorted on a FACS Aria (BD Biosciences). For intracellular transcription factor staining, PBMCs were treated with Transcriptional Factor Fixation/ Permeabilization kit (ebioscience). FACS analyses were performed on a BD LSR II flow cytometer (BD Biosciences) and analyzed using FlowJo software (Tree Star, Inc). Antibody details in S3 Table.

### Co-culture of B and T cell subsets

PBMCs were FACS-sorted into PD-1^+^CXCR3^+^CXCR5^+^CD4^+^ (Tfh-1 cells), PD-1^+^CXCR3^-^CXCR5^+^CD4^+^ (cTfh cells), PD-1^+^CXCR3^+^CXCR5^-^CD4^+^ (Th1 cells), CD4^-^CD19^+^CD21^+^CD27^-^ (naïve B cells) and CD4^-^CD19^+^CD21^+^CD27^+^ (MBCs). Each T cell subset (1.5 × 10^4^ to 5 × 10^4^) was co-cultured with naïve B cells or MBCs at ratio of 1:1 for 2, 4, 6, 8 or 12 days in complete medium with staphylococcal enterotoxin B (SEB) (1.5μg/ml; Sigma-Aldrich) in 96 round U-bottomed plates at 37°C. After co-culture, B cell number, phenotype, cytokine and Ig levels in supernatants were assessed using multiplex cytokine or isotyping assays and staining cells with anti-human monoclonal antibodies. Antibody details in S3 Table.

### PBMC stimulation with *P*. *falciparum*-infected RBC lysate

PBMCs were cultured with uninfected red blood cells (uRBCs) or *P*. *falciparum*-infected red blood cells (iRBCs). PBMCs were cultured in complete RPMI (RPMI 1640 plus 10% fetal calf serum, 1% penicillin/streptomycin) in flat-bottom 96 well plates, at 37°C in a 5% CO_2_ atmosphere. PBMCs were stimulated with iRBC or uRBC lystate in a ratio of 3 RBCs per PBMC. At day three of co-culture, cells were centrifuged and supernatants were recovered for cytokine analysis or frozen at -80 degrees.

### Measuring cytokines in plasma and supernatants of stimulated PBMCs

Plasma or supernatants were thawed and immediately analyzed with either ProcartaPlex human cytokine assays (affymetrix, ebioscience) or LEGENDplex (Biolegend) as recommended by the manufacturer. Briefly, 100 μL of plasma at 1∶2 dilution or 100 μL of supernatant were incubated with anti-cytokine antibody-coupled magnetic beads at room temperature shaking at 300 RPM in the dark. After several washes, the beads were then incubated with a biotinylated detector antibody at room temperature before incubation with streptavidin-phycoerythrin. Finally, the complexes were resuspended in 125 μL of detection buffer and 100 beads were counted with a Luminex 200 device (BioRad Laboratories, Inc.). Final concentrations were calculated from the mean fluorescence intensity and expressed in pg/mL using standard curves with known concentrations of each cytokine.

### B cell receptor cross-linking

Total B cells or naïve B cells from PBMC were negatively selected using EasySep Human B Cell Enrichment Kit (STEMCELL Technologies) as recommended by the manufacturer. Using the appropriate antibodies, the final purities of the start and isolated fractions were assessed. The purity of the final fraction was approximately 98%.

For PBMC supernatant-B cell stimulation, B cell receptors were cross-linked with 1–2μg/mL of anti-IgM (for naïve B cells only) or 2μg/mL each of anti-λ/κ light chains (for total B cells) in the presence of PBMC, PBMC/uRBC, PBMC/iRBC or iRBC lysate only supernatant. For cytokine blocking experiments, anti-cytokine neutralizing or receptor blocking antibodies were added either before or during the addition of supernatant. B cell receptors were also cross-linked in the presence of different concentrations of recombinant cytokines. For tonsillar B cell studies, total B cells negatively isolated from tonsils were stimulated with 2μg/mL each of anti-λ/κ light chains in the presence or absence of recombinant human IFN-γ. Staining with anti-CD10, IgD and CXCR4, identified the different B cell subsets.

### BCR crosslinking and phosphorylation of signaling and adaptor proteins

Whole PBMCs were thawed and plated on a 96 well plate with 2 × 10^6^ cells of each donor per well, and stained for CD10, CD19, CD20 CD21, CD27 and FCRL5 (509f6) (Biolegend) at 4°C in 4% PBS-FBS for 20 min. Cells were washed in 0.5% PBS-BSA and incubated at 37°C for 30 min before adding F(ab')2 anti-IgM and anti-IgG (Southern Biotech, Birmingham, AL and Jackson ImmunoResearch, respectively) at a final concentration of 10 μg/ml and incubating at 37°C for 5 min. For the detection of phospho-proteins by flow cytometry, cells were fixed and permeabilized according to the manufacturer's protocol using the FoxP3 Staining Buffer Set (eBioscience). Cells were then stained with antibodies specific for phospho-Syk (Y352) (pSyk) and phospho-PI3Kinase p85 (Y458)/p55 (Y199) (pPI3K) and T-bet. Antibody details in S3 Table.

### Statistical analysis

Continuous data were compared using the paired or unpaired Student's T-test and ANOVA. Bonferroni adjustments (T-tests) and Sidak adjustments (ANOVA) were applied to correct for multiple comparisons where appropriate. Correlations were calculated with Pearson correlation coefficient and their significance was determined using Fisher’s Z-test. All analyses were performed in Prism 6.0e (GraphPad Software) or R 3.1.2 [53].

## Supporting information

S1 Fig Total IgG, IgG1 and IgG3 surface expression on mature B cells from Malian children (n = 9) and U.S. (n = 9) adults (top, representative individuals). p values were determined by paired Student’s *t* test with Bonferroni adjustments. ****P<0.0001, ***P<0.001, **P<0.01, *P<0.05, ns = not significant.
(TIF)

S2 Fig Flow cytometry analysis showing correlation between T-bet expression (MFI) and (**A**) IgG1, (**B**) IgG3 and (**C**) total IgG surface expression on B cell subsets of Malian children (n = 10). Pearson correlation were used for correlative analyses.
(TIF)

S3 Fig Total IgG1, IgG2, IgG3 and IgG4 determined by bead-based multiplex assay at healthy baseline (HB) before malaria and during acute febrile malaria (Mal) in plasma of Malian children (n = 19). p values were determined by paired Student’s *t* test with Bonferroni adjustments.
(TIF)

S4 Fig PBMCs of healthy U.S. adults (n = 5) were stimulated in vitro with *P. falciparum*-infected red blood cell (iRBC) lysate or uninfected red blood cell (uRBC) lysate for 3 days. The resulting supernatants or the iRBC lysate alone were transferred to PBMCs from the same U.S. adults (n = 5) in the presence of media alone, anti-IgM, anti-CD40, or both, followed by staining for CD69, CD86, CD10, CD19 and IgD. Fold change in (**A**) CD69 and (**B**) CD86 MFI in stimulated naïve B cells relative to unstimulated naïve B cells (right, representative histograms). p values were determined by paired Student’s *t* test with Bonferroni adjustments where appropriate. ****P<0.0001, ***P<0.001, **P<0.01, *P<0.05, ns = not significant.
(TIF)

S5 Fig PBMCs of healthy U.S. adults (n = 8) were stimulated in vitro with iRBC lysate or uRBC lysate for 3 days. The concentrations of twenty cytokines in the resulting supernatants were determined by a bead-based multiplex assay. Shown are fold changes in concentrations of cytokines in supernatants of PBMCs stimulated with iRBCs versus uRBCs. p values were determined by paired Student’s *t* test with Bonferroni adjustments where appropriate. ****P<0.0001, ***P<0.001, **P<0.01, *P<0.05, ns = not significant.
(TIF)

S6 Fig PBMCs of healthy U.S. adults (n = 8) were stimulated in vitro with iRBC lysate, uRBC lysate or media alone for 3 days (same experiment as S5 Fig). Cytokine concentrations in the resulting supernatants were determined by a bead-based multiplex assay. Shown are absolute concentrations of cytokines in supernatants. p values were determined by paired Student’s *t* test with Bonferroni adjustments where appropriate. ****P<0.0001, ***P<0.001, **P<0.01, *P<0.05, ns = not significant.
(TIF)

S7 Fig Gating strategy for FACS-sorted PBMCs from healthy U.S. adults into naïve B cells (CD19+CD21+IgD+), cTfh cells (CD4+PD-1+CXCR5+CXCR3-), Tfh-1 cells (CD4+PD-1+CXCR5+CXCR3+) and Th-1 cells (CD4+PD-1+CXCR5-CXCR3+). (TIF)

S8 Fig PBMCs of U.S. adults were FACs-sorted into naïve B cells (CD19+CD21+IgD+), cTfh cells (CD4+PD-1+CXCR5+CXCR3-), Tfh-1 cells (CD4+PD-1+CXCR5+CXCR3+) and Th-1 cells (CD4+PD-1+CXCR5-CXCR3+). Autologous naïve B cells were cultured for 2 days with each T cell subset in the presence of SEB, or with SEB alone. (**A**) Percentage viable B cells over 8 days of co-culture. Fold change in MFI on B cells at 2 days for (**B**) CD69, (**C**) BLIMP-1 and (**D**) AID, relative to SEB alone control. A and B from same experiment (n = 15); C and D from same experiment (n = 5). p values were determined by paired Student’s *t* test with Bonferroni adjustments where appropriate. ****P<0.0001, ***P<0.001, **P<0.01, *P<0.05, ns = not significant.
(TIF)

S9 Fig Percentage of T-bet^hi^ CD19+CD21+IgD+ B cells after 2 days of co-culture with indicated T cell subsets with SEB alone or with SEB plus recombinant human IFN-γ. p values were determined by paired Student’s t test with Bonferroni adjustments where appropriate. ****P<0.0001, ***P<0.001, **P<0.01, *P<0.05, ns = not significant.
(TIF)

S10 Fig (**A**) Class switching of CD19+CD21+IgD+ B cells cultured with c-Tfh cells, Tfh-1 cells or Th1 cells over 12 days. (**B**) Secreted antibody production by CD19+CD21+IgD+ B cells cultured with c-Tfh cells, Tfh-1 cells or Th1 cells over 12 days. p values were determined by paired Student’s *t* test with Bonferroni adjustments where appropriate. ****P<0.0001, ***P<0.001, **P<0.01, *P<0.05, ns = not significant.
(TIF)

S1 Table Demographic and clinical data of study subjects used for gene expression microarray analysis. (TIF)

S2 Table Demographic and clinical data of study subjects whose PBMCs were used to characterize T-bet expression in B cells. (TIF)

S3 Table Antibodies used for T and B cell characterization, intracellular staining and sorting. (TIF)
