# Supplementary figures and images for: Malaria-induced interferon-γ drives the expansion of Tbethi atypical memory B cells
Source: PLoS Pathog. 2017 Sep 27;13(9):e1006576. doi: 10.1371/journal.ppat.1006576 (PMC5633206; doi:10.1371/journal.ppat.1006576)

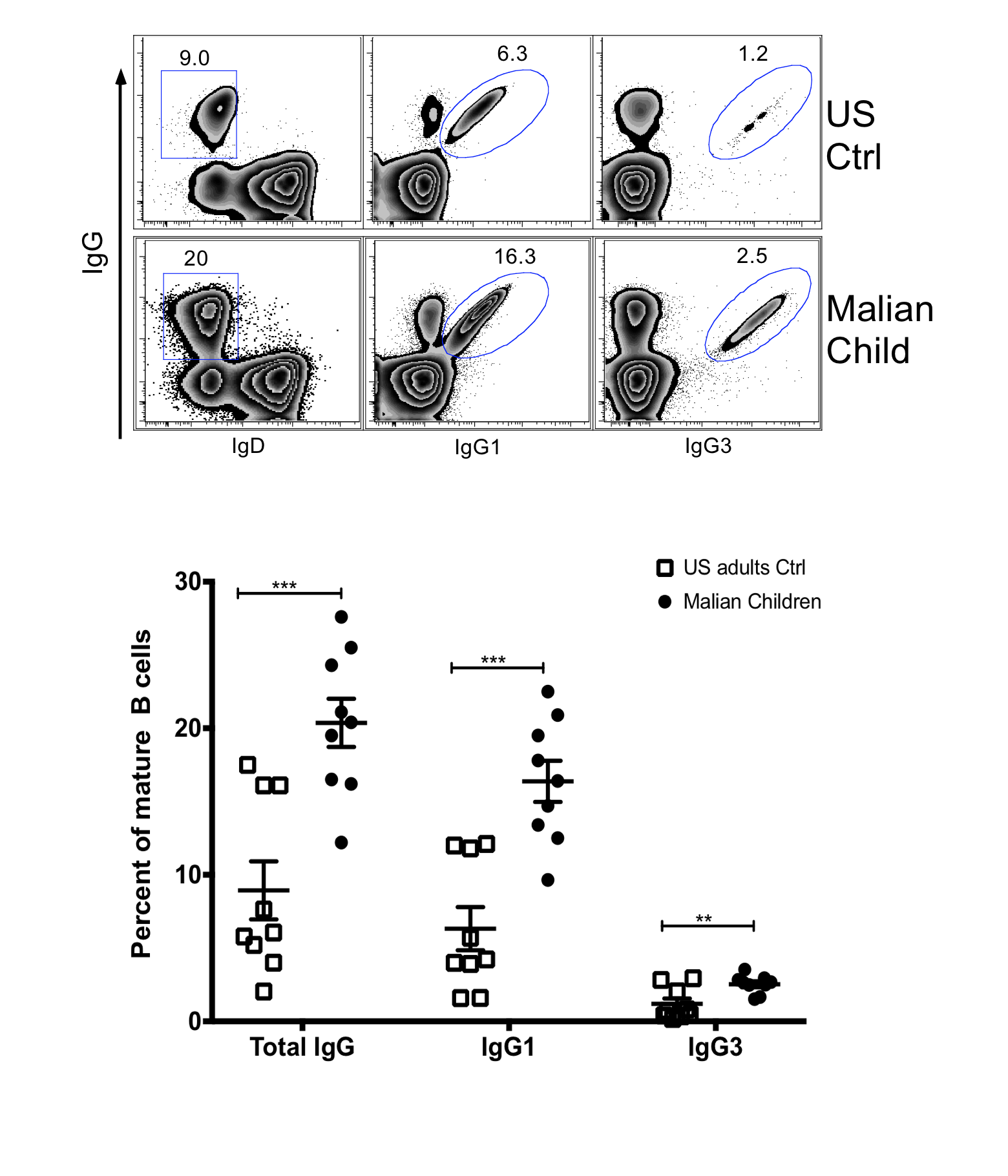

Supplement: S1 Fig — p values were determined by paired Student’s t test with Bonferroni adjustments. ****P<0.0001, ***P<0.001, **P<0.01, *P<0.05, ns = not significant. (TIF) [file ppat.1006576.s001.tif]

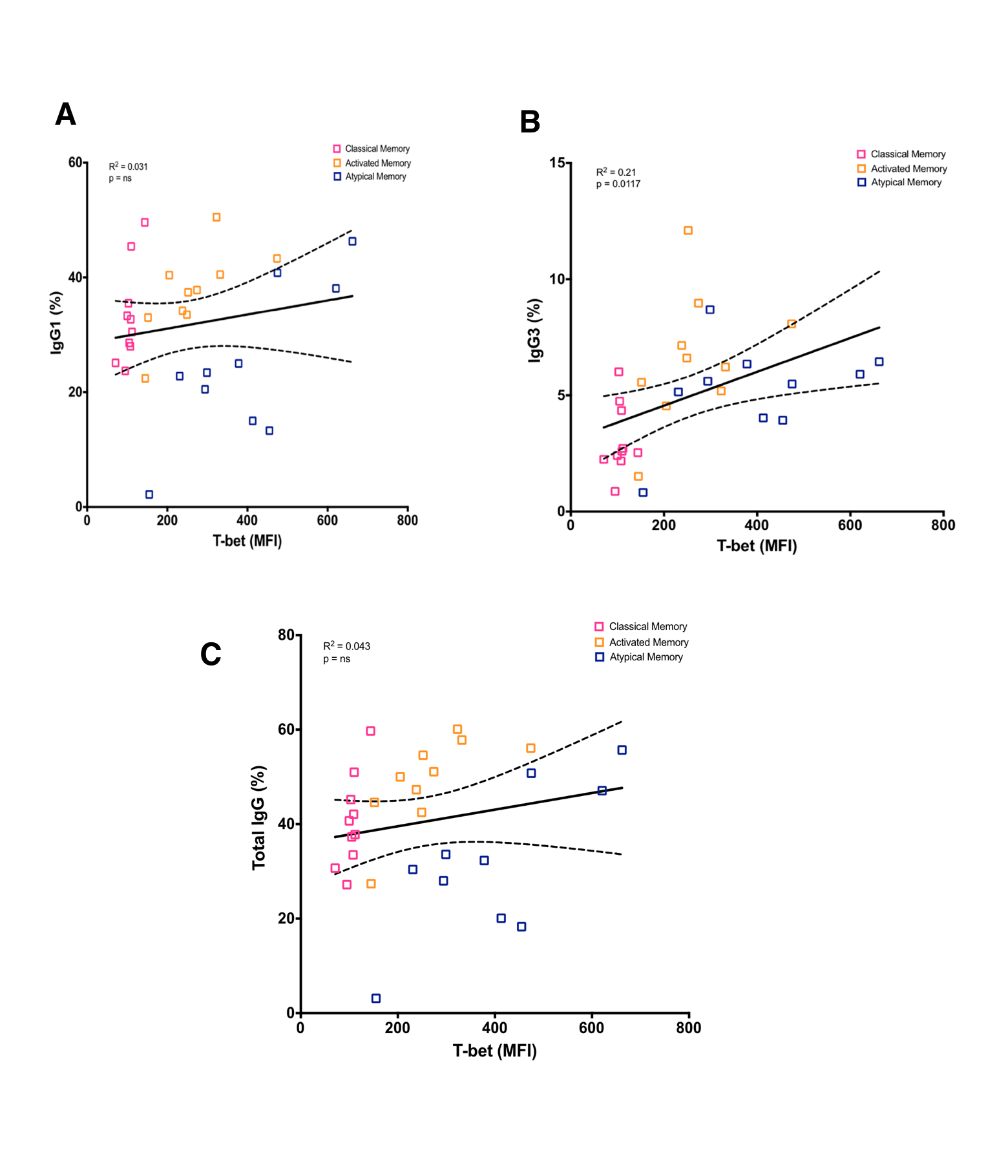

Supplement: S2 Fig — Flow cytometry analysis showing correlation between T-bet expression (MFI) and (A) IgG1, (B) IgG3 and (C) total IgG surface expression on B cell subsets of Malian children (n = 10). Pearson correlation were used for correlative analyses. (TIF) [file ppat.1006576.s002.tif]

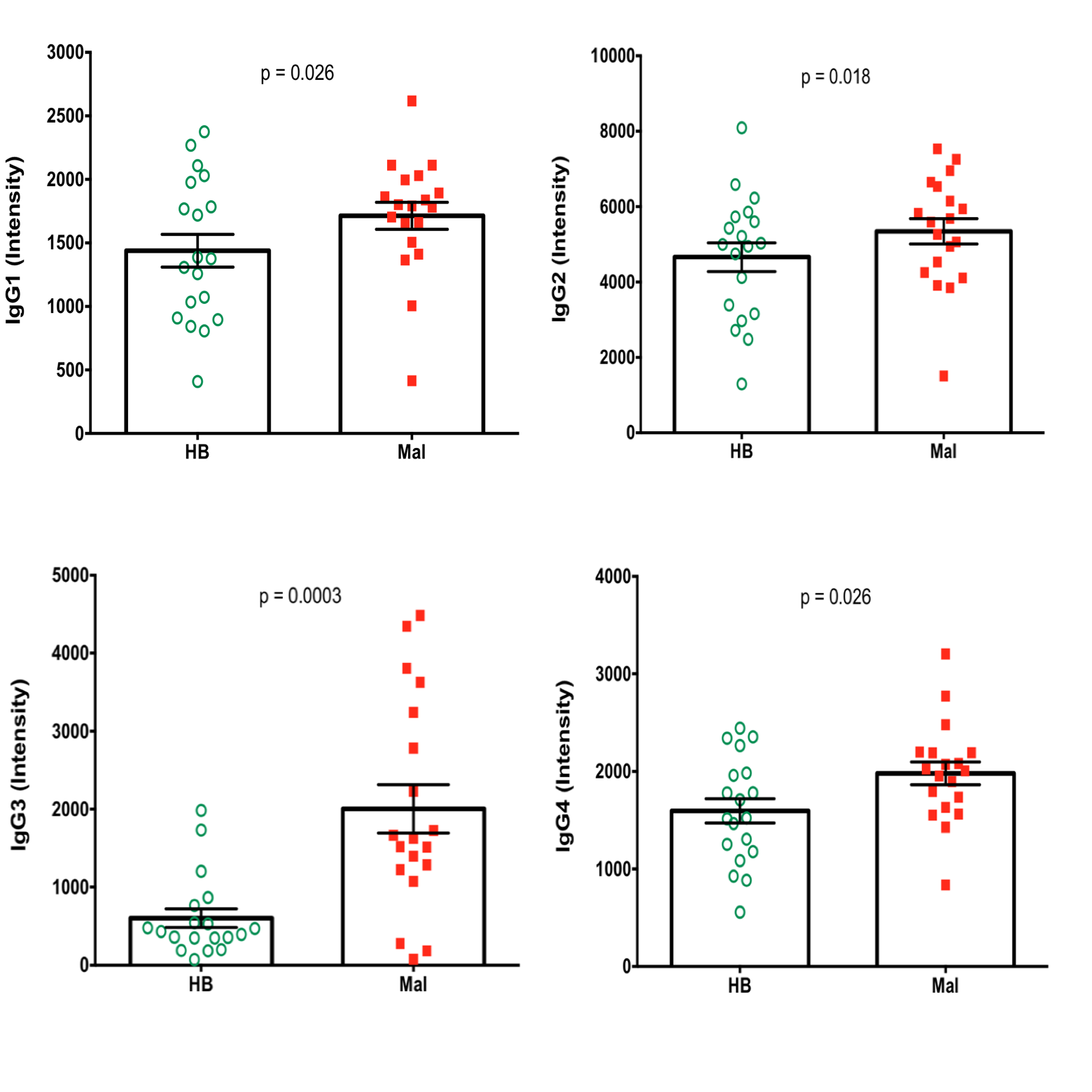

Supplement: S3 Fig — p values were determined by paired Student’s t test with Bonferroni adjustments. (TIF) [file ppat.1006576.s003.tif]

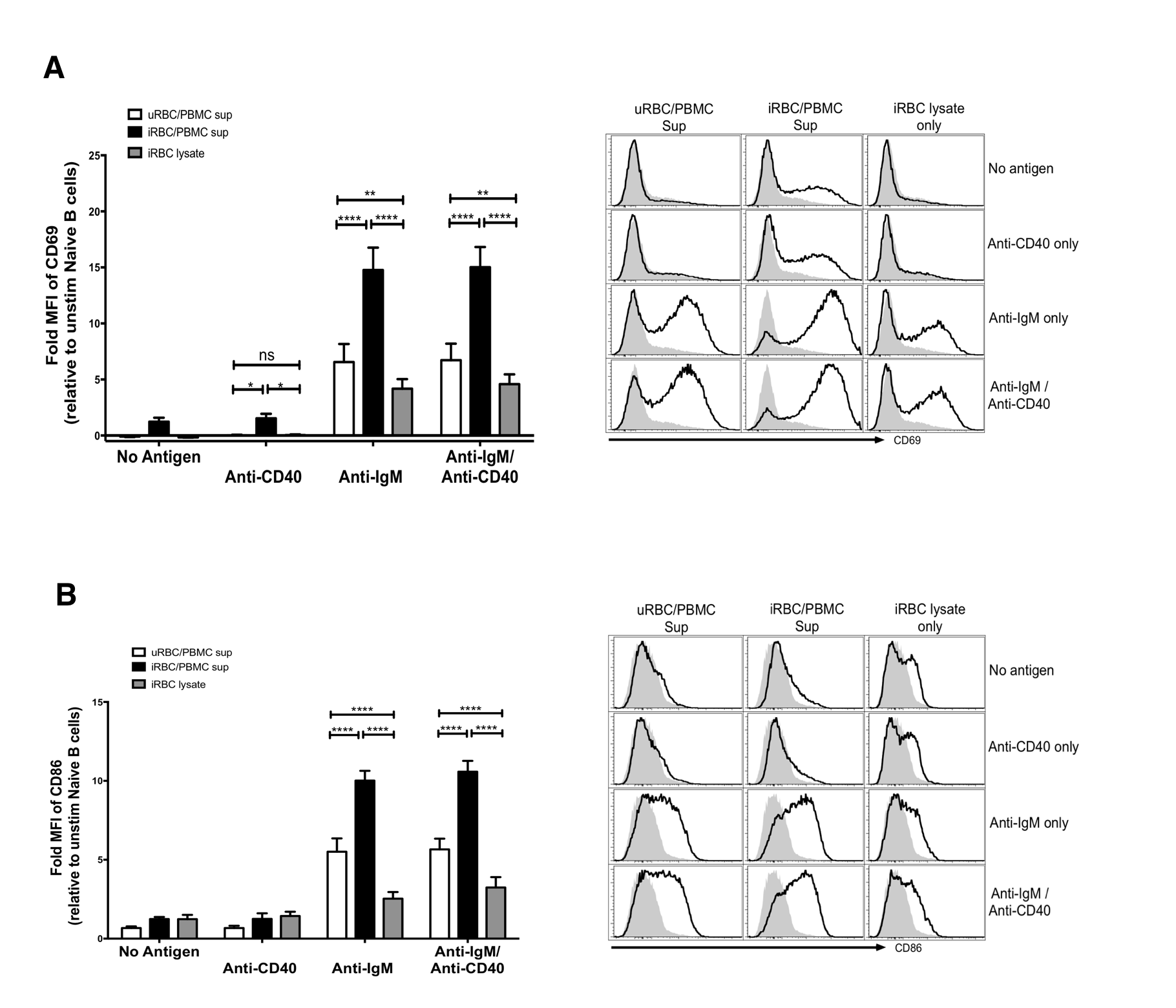

Supplement: S4 Fig — The resulting supernatants or the iRBC lysate alone were transferred to PBMCs from the same U.S. adults (n = 5) in the presence of media alone, anti-IgM, anti-CD40, or both, followed by staining for CD69, CD86, CD10, CD19 and IgD. Fold change in (A) CD69 and (B) CD86 MFI in stimulated naïve B cells relative to unstimulated naïve B cells (right, representative histograms). p values were determined by paired Student’s t test with Bonferroni adjustments where appropriate. ****P<0.0001, ***P<0.001, **P<0.01, *P<0.05, ns = not significant. (TIF) [file ppat.1006576.s004.tif]

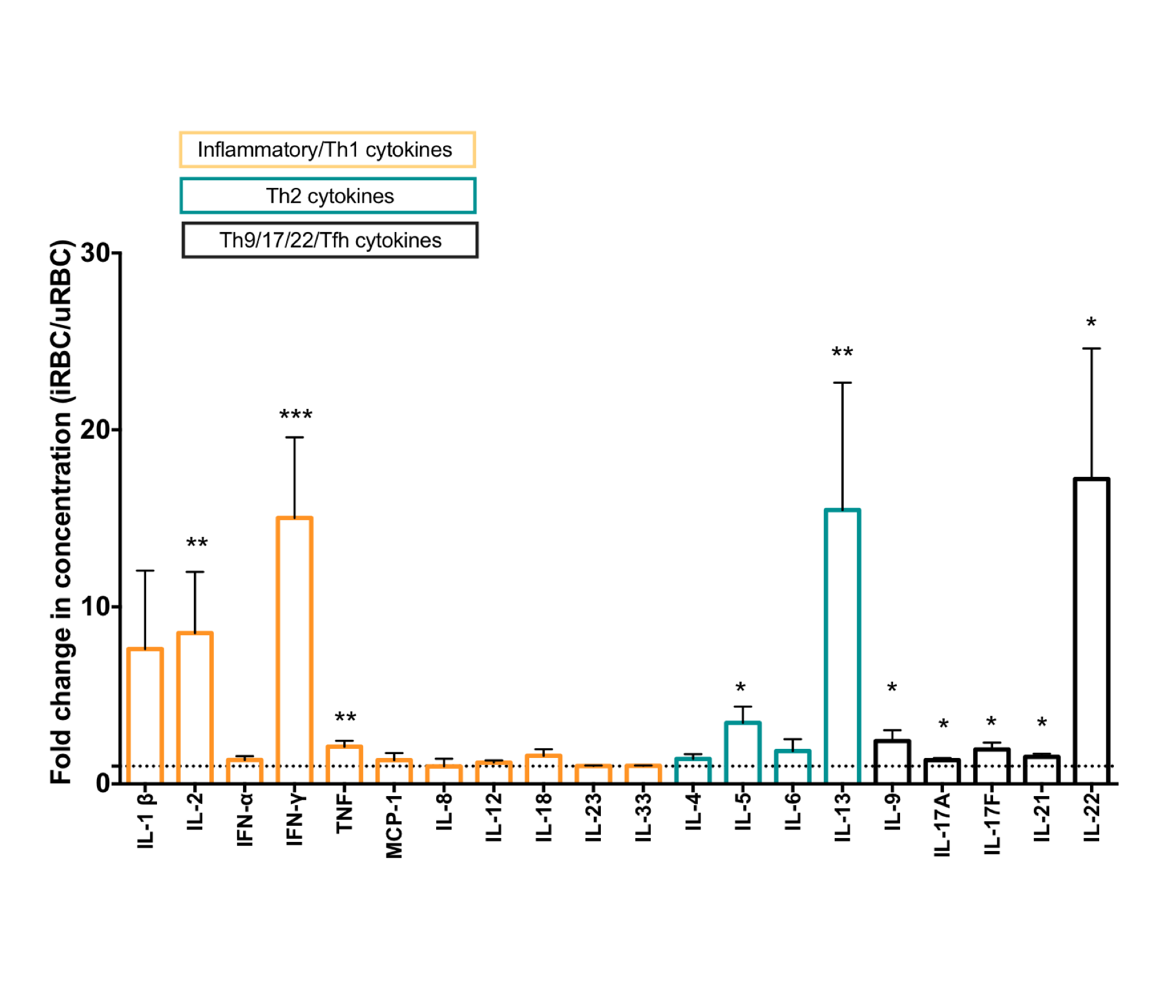

Supplement: S5 Fig — The concentrations of twenty cytokines in the resulting supernatants were determined by a bead-based multiplex assay. Shown are fold changes in concentrations of cytokines in supernatants of PBMCs stimulated with iRBCs versus uRBCs. p values were determined by paired Student’s t test with Bonferroni adjustments where appropriate. ****P<0.0001, ***P<0.001, **P<0.01, *P<0.05, ns = not significant. (TIF) [file ppat.1006576.s005.tif]

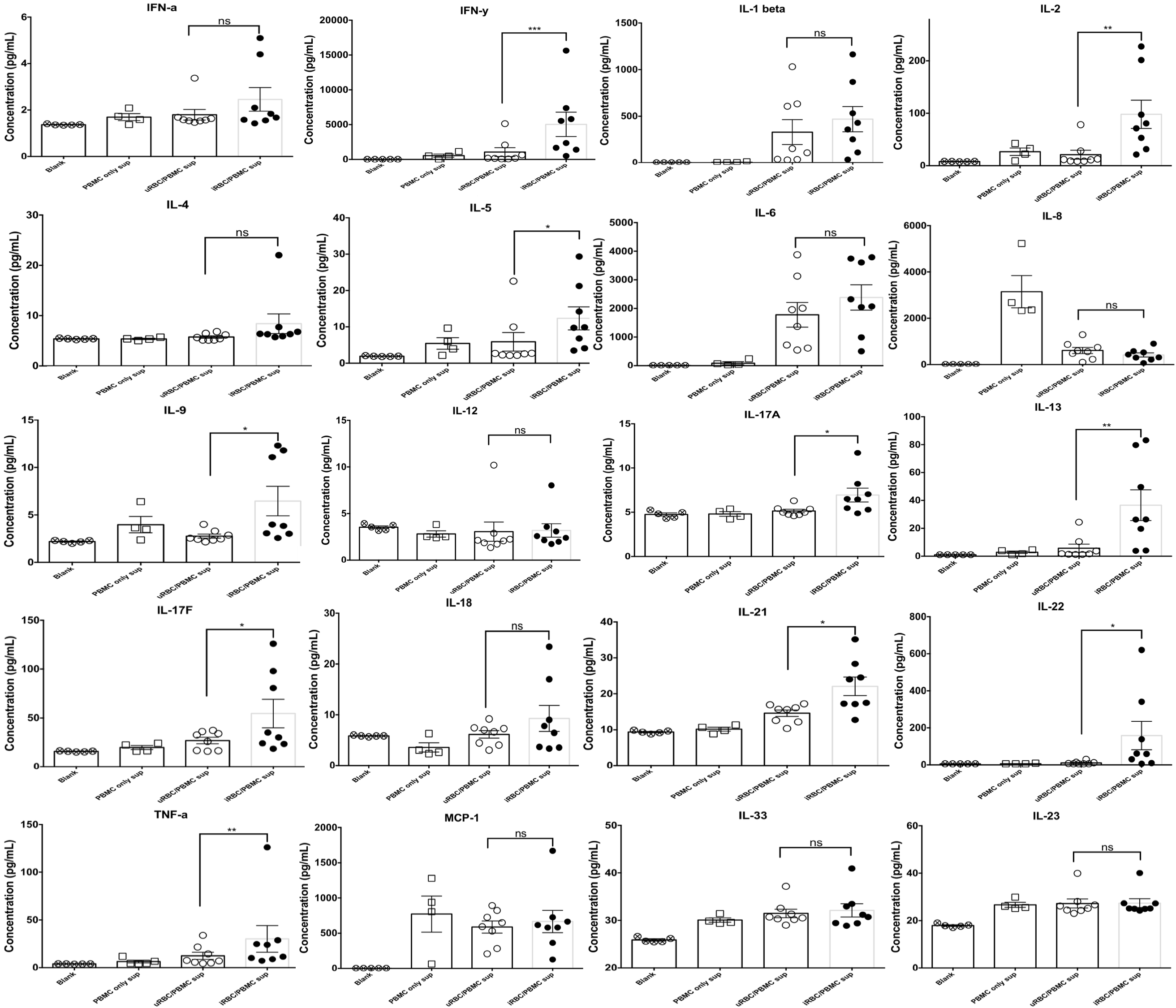

Supplement: S6 Fig — Cytokine concentrations in the resulting supernatants were determined by a bead-based multiplex assay. Shown are absolute concentrations of cytokines in supernatants. p values were determined by paired Student’s t test with Bonferroni adjustments where appropriate. ****P<0.0001, ***P<0.001, **P<0.01, *P<0.05, ns = not significant. (TIF) [file ppat.1006576.s006.tif]

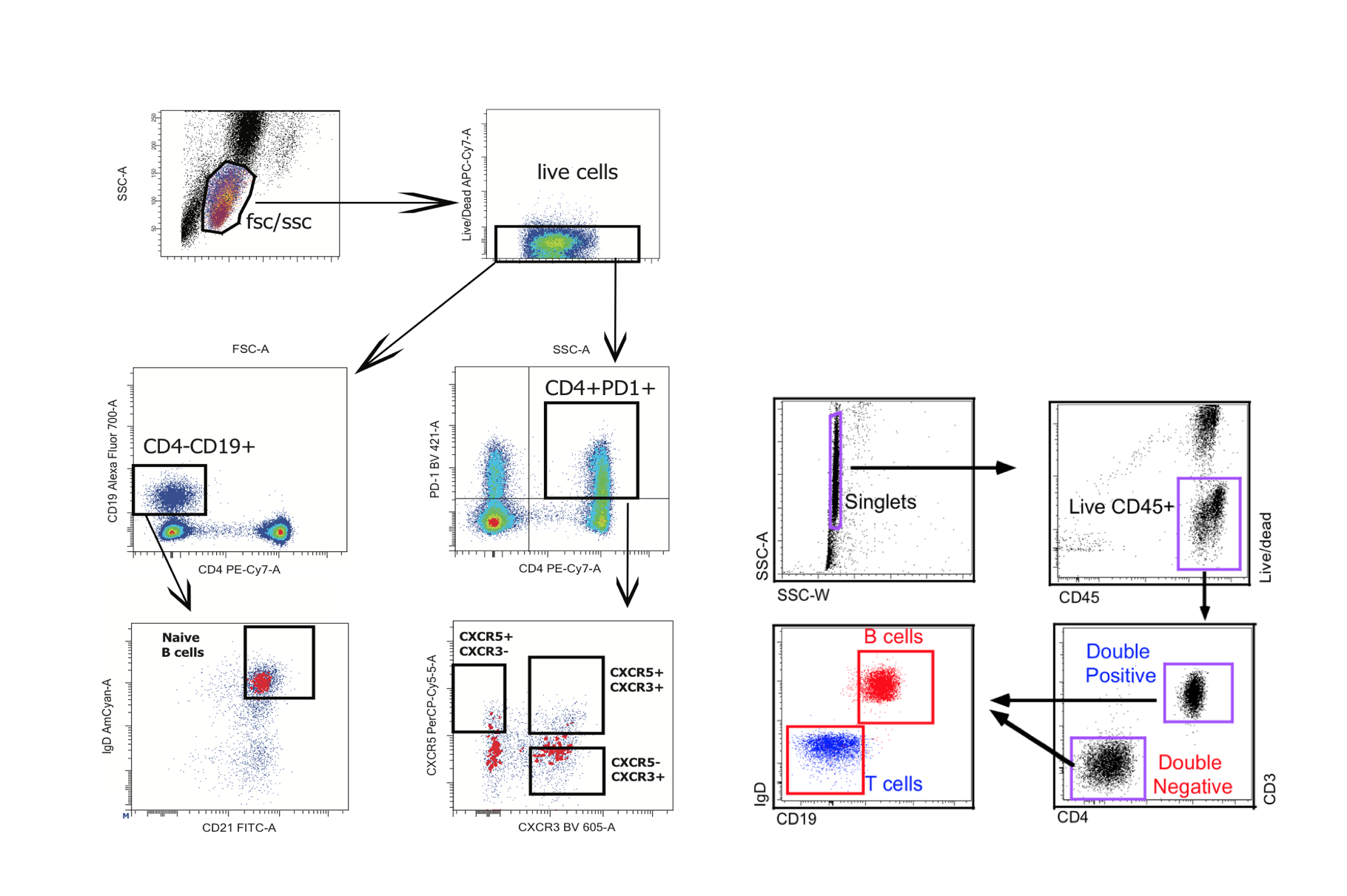

Supplement: S7 Fig — (TIF) [file ppat.1006576.s007.tif]

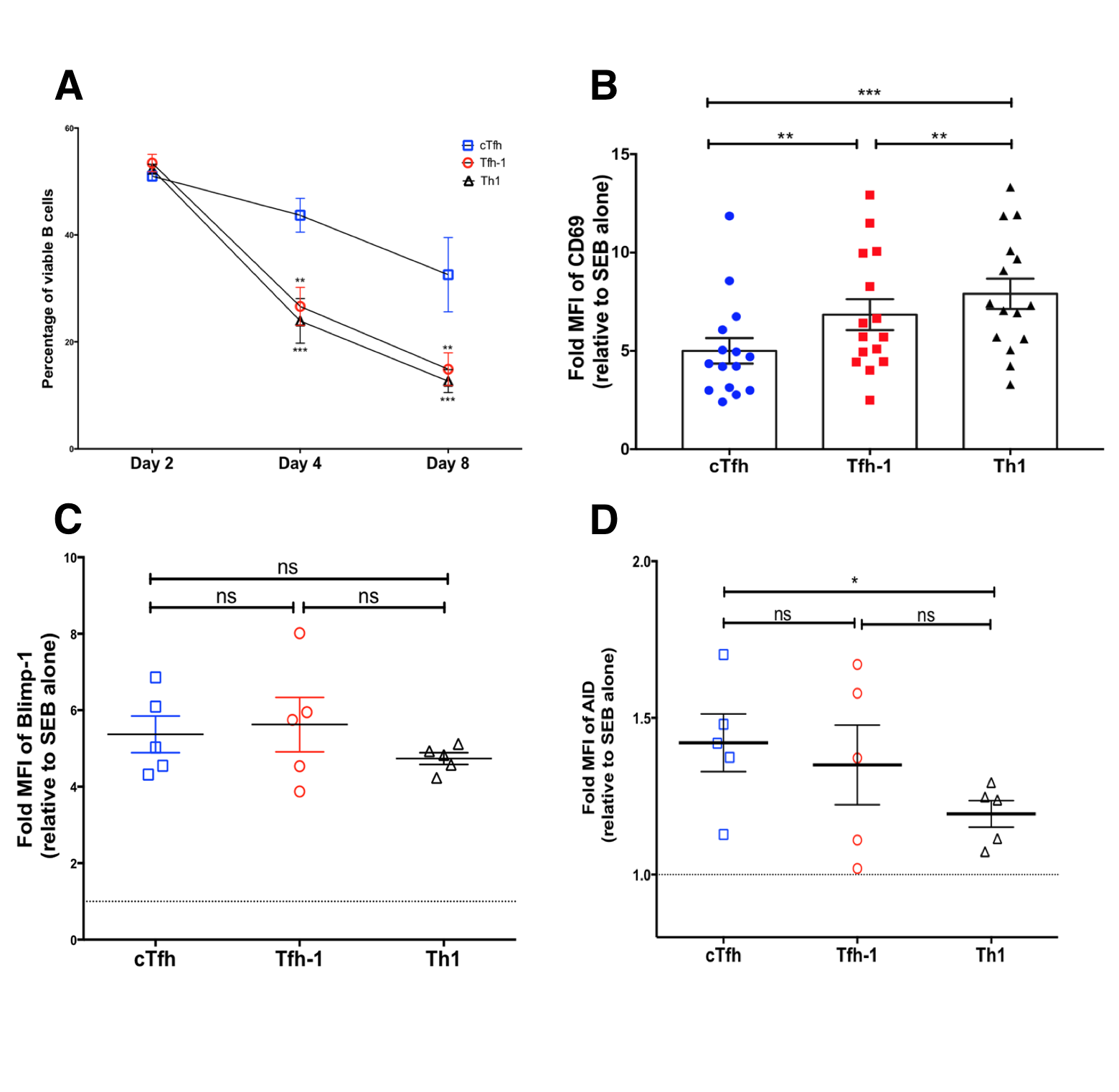

Supplement: S8 Fig — Autologous naïve B cells were cultured for 2 days with each T cell subset in the presence of SEB, or with SEB alone. (A) Percentage viable B cells over 8 days of co-culture. Fold change in MFI on B cells at 2 days for (B) CD69, (C) BLIMP-1 and (D) AID, relative to SEB alone control. A and B from same experiment (n = 15); C and D from same experiment (n = 5). p values were determined by paired Student’s t test with Bonferroni adjustments where appropriate. ****P<0.0001, ***P<0.001, **P<0.01, *P<0.05, ns = not significant. (TIF) [file ppat.1006576.s008.tif]

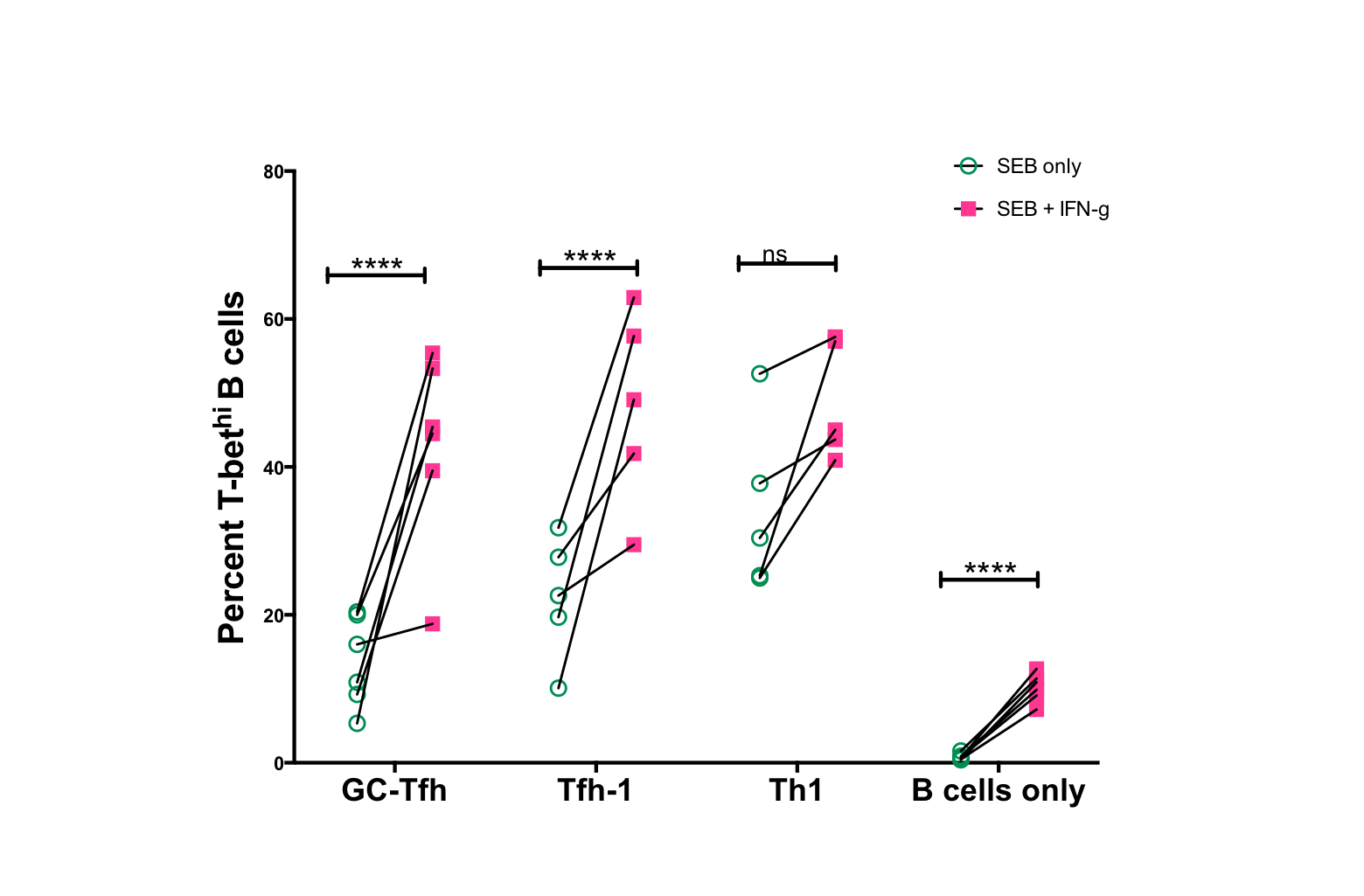

Supplement: S9 Fig — ****P<0.0001, ***P<0.001, **P<0.01, *P<0.05, ns = not significant. (TIF) [file ppat.1006576.s009.tif]

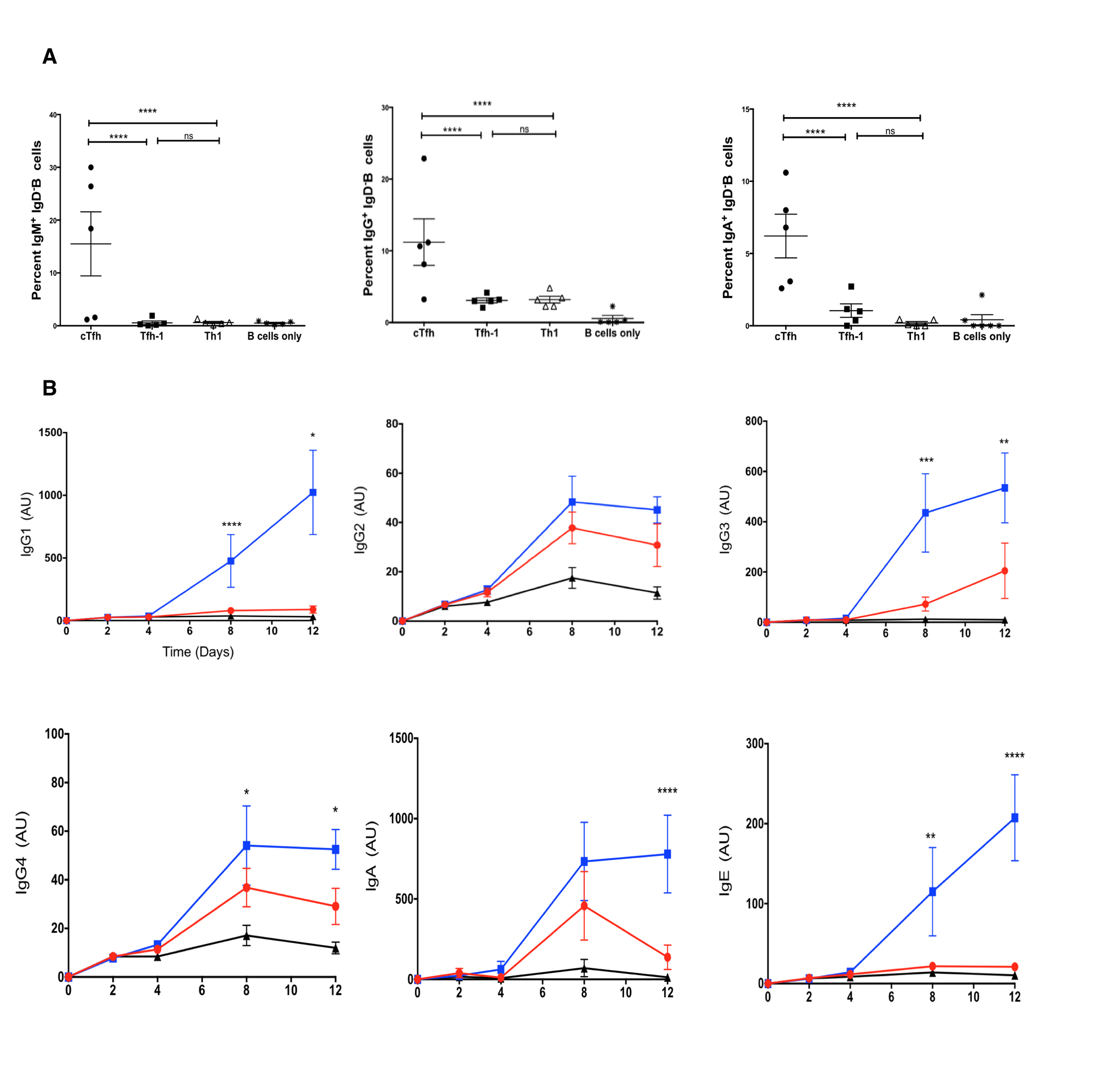

Supplement: S10 Fig — (A) Class switching of CD19+CD21+IgD+ B cells cultured with c-Tfh cells, Tfh-1 cells or Th1 cells over 12 days. (B) Secreted antibody production by CD19+CD21+IgD+ B cells cultured with c-Tfh cells, Tfh-1 cells or Th1 cells over 12 days. p values were determined by paired Student’s t test with Bonferroni adjustments where appropriate. ****P<0.0001, ***P<0.001, **P<0.01, *P<0.05, ns = not significant. (TIF) [file ppat.1006576.s010.tif]

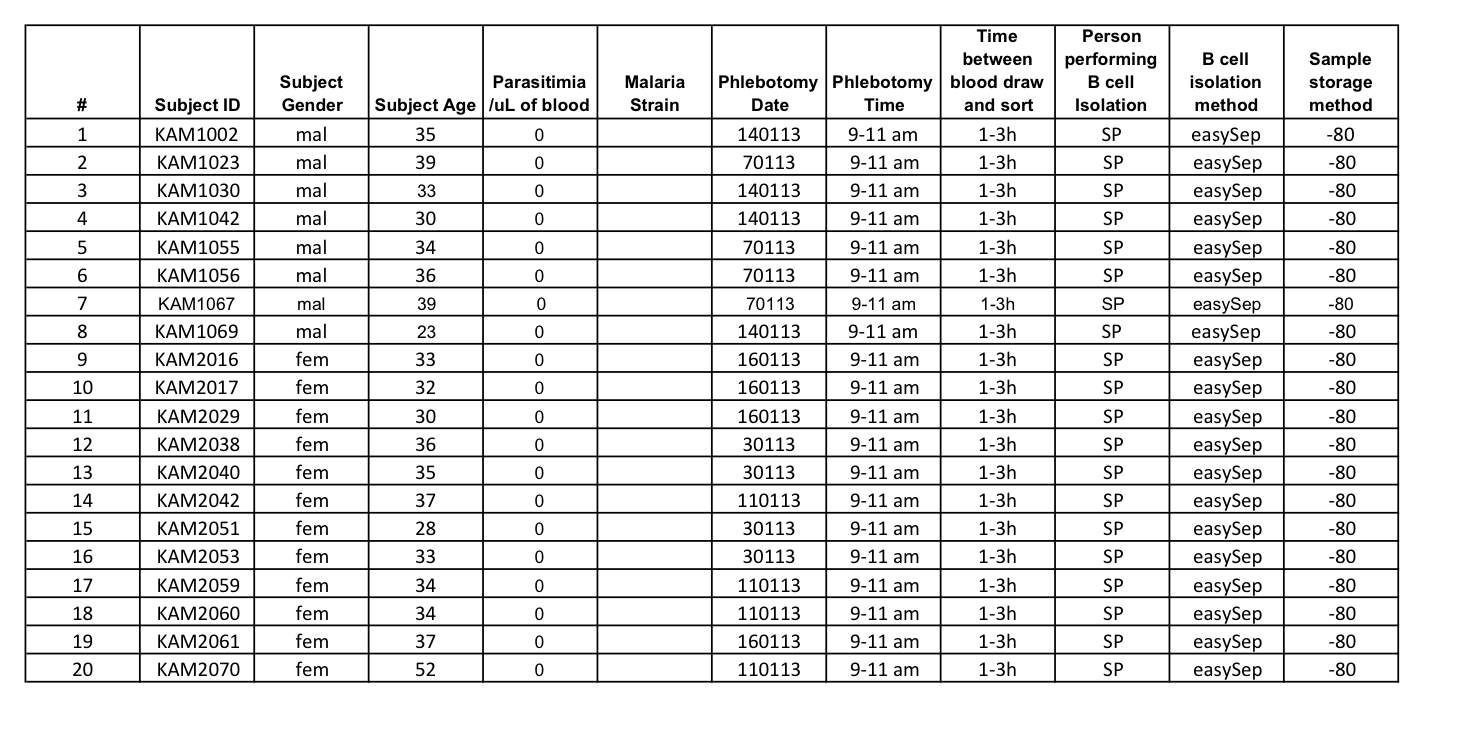

Supplement: S1 Table — (TIF) [file ppat.1006576.s011.tif]

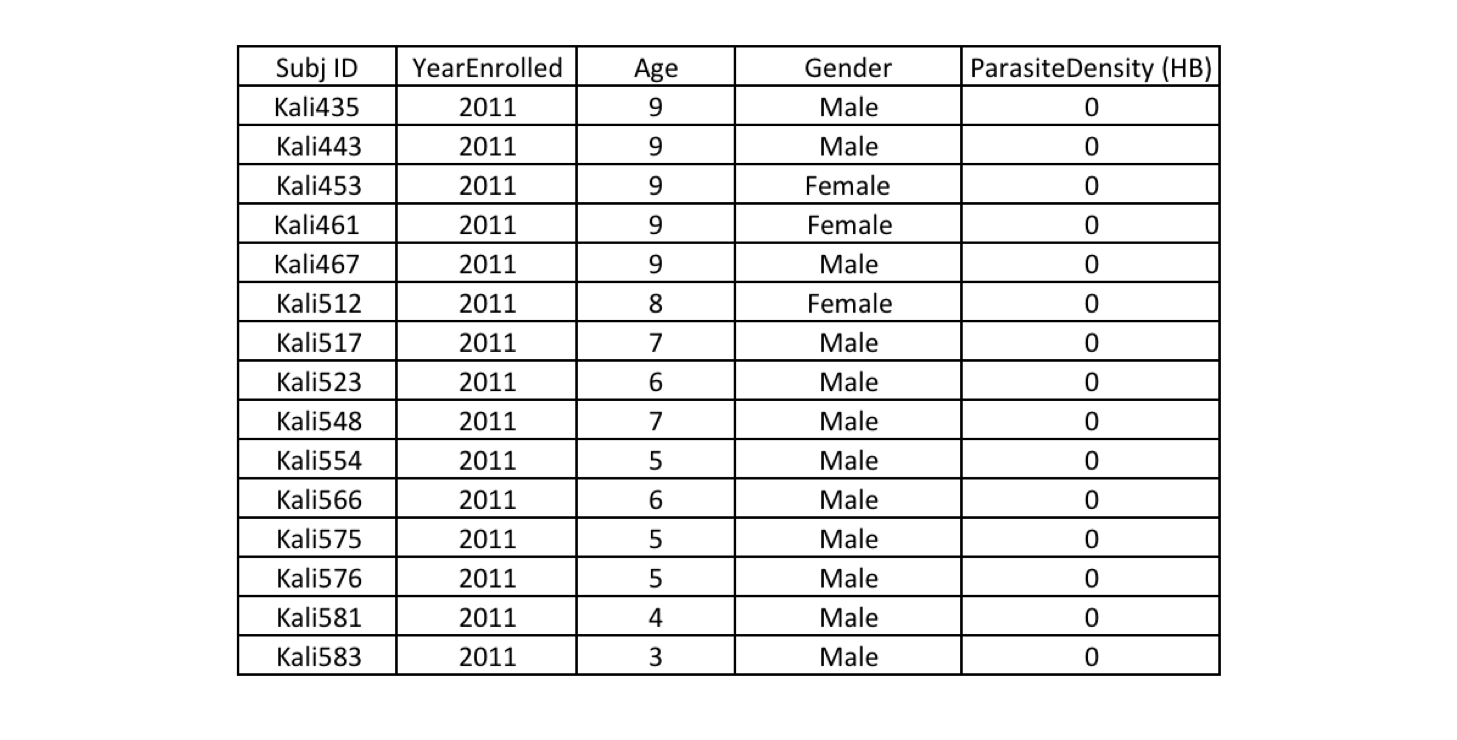

Supplement: S2 Table — (TIF) [file ppat.1006576.s012.tif]

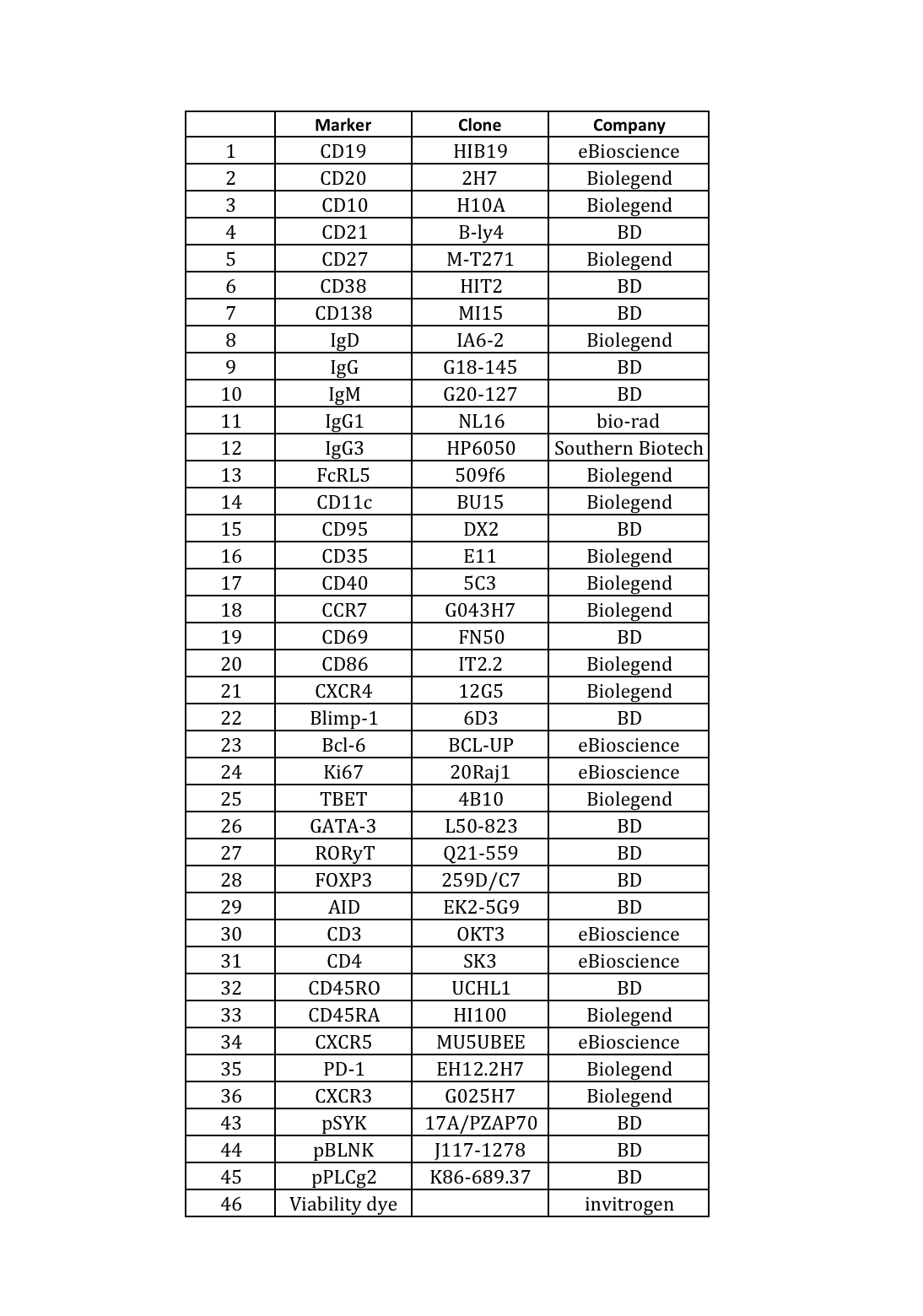

Supplement: S3 Table — (TIF) [file ppat.1006576.s013.tif]
